# Assessment of human emotional reactions to visual stimuli “deep-dreamed” by artificial neural networks

**DOI:** 10.3389/fpsyg.2024.1509392

**Published:** 2024-12-24

**Authors:** Agnieszka Marczak-Czajka, Timothy Redgrave, Mahsa Mitcheff, Michael Villano, Adam Czajka

**Affiliations:** ^1^Department of Computer Science and Engineering, University of Notre Dame, Notre Dame, IN, United States; ^2^Department of Psychology, University of Notre Dame, Notre Dame, IN, United States

**Keywords:** emotional reactions, visual stimuli, deep learning, artificial neural networks, visual stimuli synthesis

## Abstract

**Introduction:**

While the fact that visual stimuli synthesized by Artificial Neural Networks (ANN) may evoke emotional reactions is documented, the precise mechanisms that connect the strength and type of such reactions with the ways of how ANNs are used to synthesize visual stimuli are yet to be discovered. Understanding these mechanisms allows for designing methods that synthesize images attenuating or enhancing selected emotional states, which may provide unobtrusive and widely-applicable treatment of mental dysfunctions and disorders.

**Methods:**

The Convolutional Neural Network (CNN), a type of ANN used in computer vision tasks which models the ways humans solve visual tasks, was applied to synthesize (“dream” or “hallucinate”) images with no semantic content to maximize activations of neurons in precisely-selected layers in the CNN. The evoked emotions of 150 human subjects observing these images were self-reported on a two-dimensional scale (arousal and valence) utilizing self-assessment manikin (SAM) figures. Correlations between arousal and valence values and image visual properties (e.g., color, brightness, clutter feature congestion, and clutter sub-band entropy) as well as the position of the CNN's layers stimulated to obtain a given image were calculated.

**Results:**

Synthesized images that maximized activations of some of the CNN layers led to significantly higher or lower arousal and valence levels compared to average subject's reactions. Multiple linear regression analysis found that a small set of selected image global visual features (hue, feature congestion, and sub-band entropy) are significant predictors of the measured arousal, however no statistically significant dependencies were found between image global visual features and the measured valence.

**Conclusion:**

This study demonstrates that the specific method of synthesizing images by maximizing small and precisely-selected parts of the CNN used in this work may lead to synthesis of visual stimuli that enhance or attenuate emotional reactions. This method paves the way for developing tools that stimulate, in a non-invasive way, to support wellbeing (manage stress, enhance mood) and to assist patients with certain mental conditions by complementing traditional methods of therapeutic interventions.

## 1 Introduction

Approximately half of our brain tissue receives visual information (Sells and Fixott, 1957), and more neurons in our brain deal with visual tasks than with the other four senses combined. Hence, humans are fundamentally visual creatures, and many aspects of our behaviour are determined by how visual information is processed by the brain, either consciously or unconsciously. Stimulation of emotions is one of the research areas studied intensively, and various visual tools have been proposed to date to aid psychologists in affective research by inducing emotional states through the presentation of images (Marchewka et al., 2014; Dan-Glauser and Scherer, 2011; Kurdi et al., 2017; Lang P. J. et al., 2008). With the recent explosion of deep learning-oriented research (LeCun et al., 2015), in particular involving convolutional neural networks (LeCun and Bengio, 1998) (CNN), which are currently used to build modern generative models (Goodfellow et al., 2014), research questions arise around functional similarities between modern CNNs and a human brain (especially the ventral stream). If such similarities exist, do they allow the construction of visual stimuli-based mechanisms for precise stimulation of emotions, with an overarching goal to apply them in mental health healing processes? The study presented in this paper contributes to a wider family of studies that attempt to answer this question.

**Figure 1:**
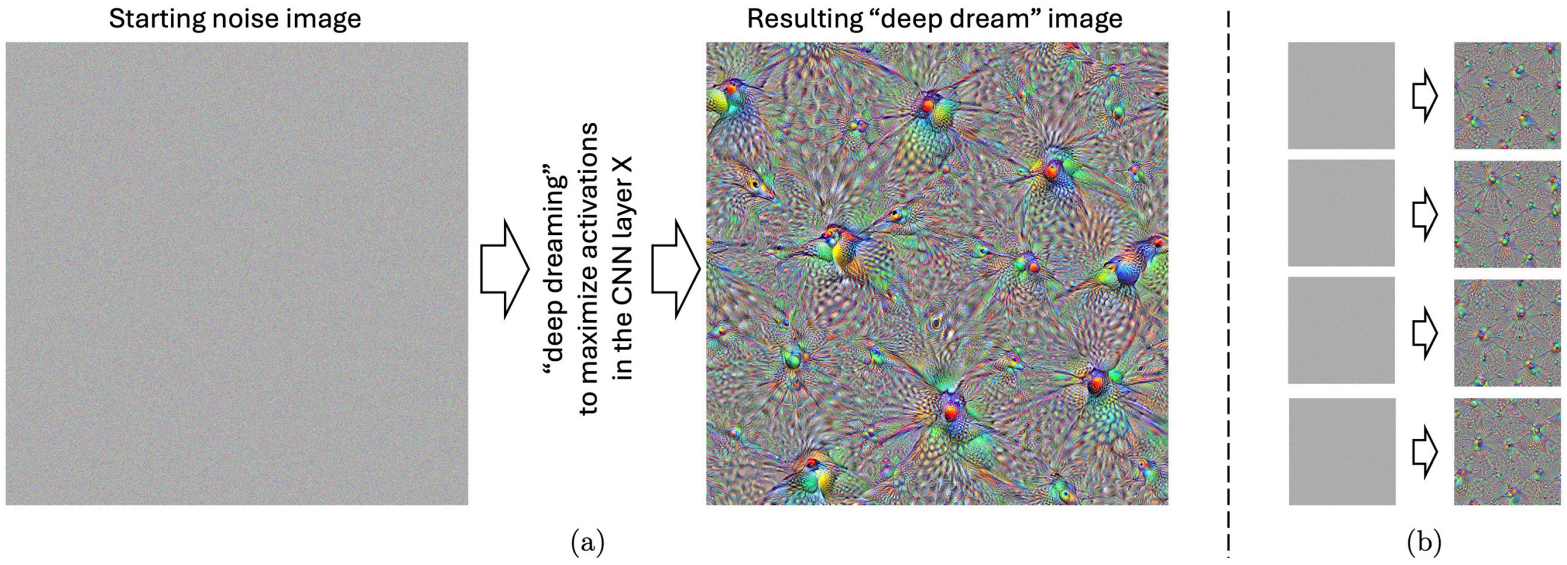
**(A)** An example of the “deep-dreamed” image, starting from a white noise image illustrated on the left. This particular image evoked the highest, on average, arousal and valence among the subjects participating in the experiment. **(B)** Four other images “deep dreamed” for that same layer, starting with different noise images. Such images were generated for all 144 layers of the Inception CNN model and utilized in this study.

In 2015, Google researchers demonstrated a visualization method that gave insights into what each section (e.g., a single neuron, a layer of neurons, or an arbitrary set of neurons) of a trained CNN is “interested in” when solving a visual classification task (Mordvintsev et al., 2015b; Szegedy et al., 2015). The method was based on iterative modification of an input to boost activations of these selected neurons. The resulting pictures (see an example in Figure 1) allowed researchers to visualize how the CNN “understands” the general notion of visual objects, thus this mechanism later received significant attention among researchers working on explainable artificial intelligence. Due to unnatural, dream-like patterns present in these samples, the “deep dream” term was coined for these visual stimuli (Mordvintsev et al., 2015a).

**Figure 2:**
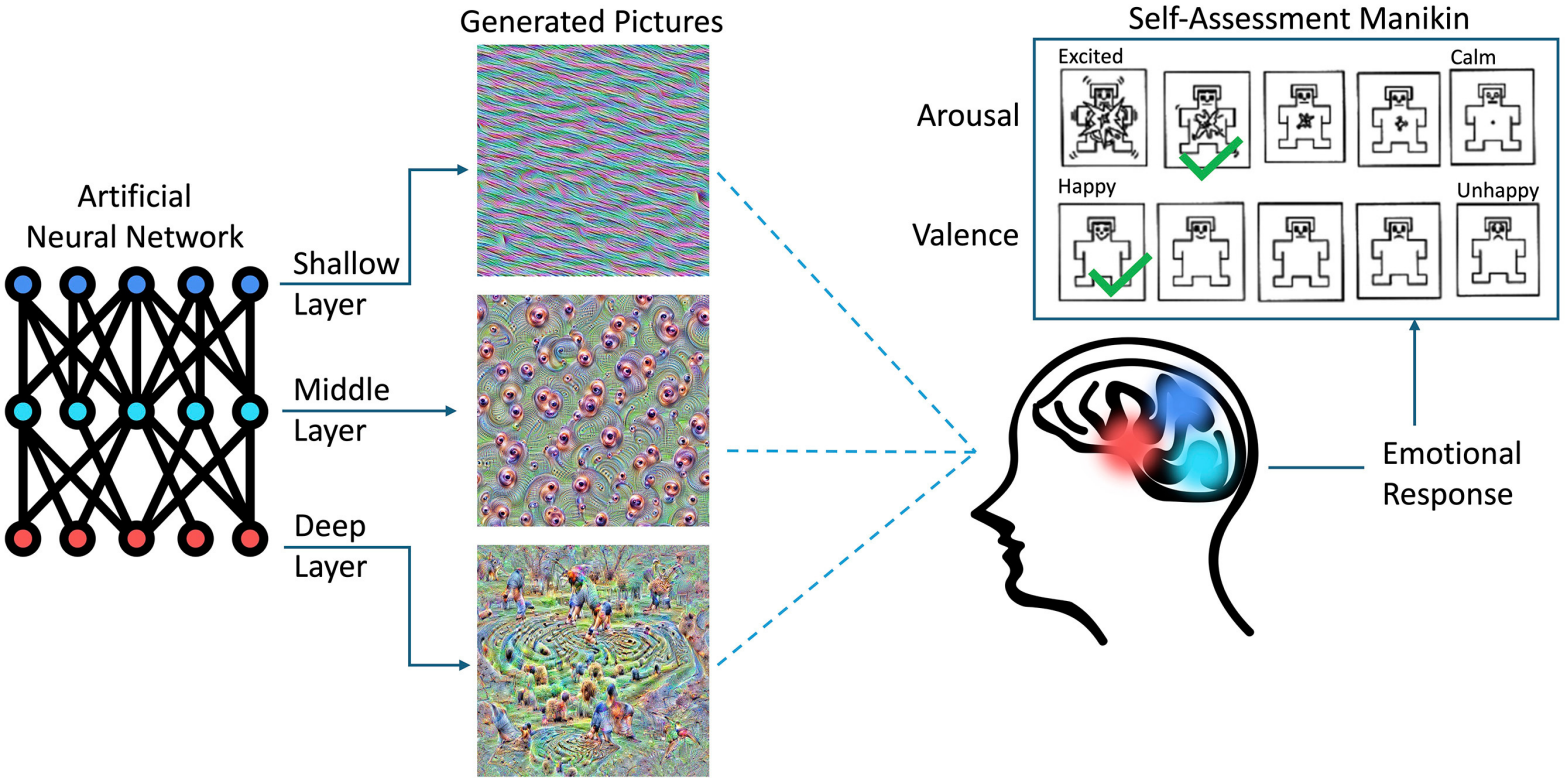
Overview of the experiment carried out in this study. The visual stimuli were synthesized in a way to activate selected sections (e.g., layers) of an Artificial Neural Network (ANN). Such stimuli, when presented to a subject, evoked reactions that were self-reported by selecting the levels of arousal and valence experienced during the experiment. The colors matching various sections of the ANN and the brain illustrate the hypothesis that visual signals synthesized that way may stimulate different functional brain areas, resulting in different emotional reactions.

This paper, to our knowledge for the first time, presents quantitative results of how humans react to pictures synthesized by a CNN, which “dream” or “hallucinate” visual inputs to maximize activations of neurons located in a given portion of the CNN model, as shown in Figure 2. This work was initially inspired by a positive feedback received from users of the LockLuck tool (described in Section 2.4), which incorporated “deep dream” pictures into psychological coaching processes, and—after presenting these unusual visuals to the coaching clients—reported an increased efficiency of the coaching sessions. In the current study, which was carried out with 150 subjects that viewed “deep dream” images carefully synthesized to boost activations of the CNN layers (one by one), we found that there is a dependency between the location of the portion of the CNN being activated and the strength of emotions self-reported by humans viewing these images.

These results are obtained on a relatively small sample of subjects, and thus should be taken with care. However, this study suggests that it is possible to use modern artificial neural networks to influence the human emotional system in a controlled way by presenting synthetically-generated rather than natural visual inputs which, in turn, could have a tremendous impact on creating effective and affordable mental health healing tools. The approach proposed in this paper is **different** from those using natural images, or recently popular creations generated by text-to-image Artificial Intelligence tools. The main difference comes from the fact of a precise control over which parts of the CNN are boosted by feeding the network with the synthesized stimuli. This approach offers an increased interpretability of the observed results and potential of mapping the CNN areas with functional regions of the brain.

## 2 Related work

### 2.1 Response to emotional visual stimuli

The emotional response to affective pictures is a complex and multidimensional phenomenon, influenced by both the content and physical properties of the stimuli. Valence and arousal are two critical dimensions used in all common emotional stimuli databases to measure these responses. Valence reflects the subjective conscious experience of feeling pleasantness or unpleasantness, while arousal relates to feelings of calmness or excitation (Barrett, 1998). These dimensions are pancultural and observable even in children as young as four to five years old (Russell and Bullock, 1985). We selected to use valence and arousal in measuring emotions in this study.

Among the most known resources providing visual stimuli are the International Affective Pictures System (IAPS) (Lang P. et al., 2008), the Nencki Affective Picture System (NAPS) (Marchewka et al., 2014), the Geneva Affective Picture Database (GAPD) (Dan-Glauser and Scherer, 2011), the Open Affective Standardized Image System (OASIS) (Kurdi et al., 2017), and EmoMadrid (Carretié et al., 2019). These databases categorize images with ratings of “low” and “high” to estimate arousal, and “positive,” “neutral,” and “negative” to estimate valence. More specifically, GAPD and NAPS are two databases that have been developed to provide a wide range of high-quality, realistic pictures for affective research. EmoMadrid further enhances the understanding of emotional responses by including low-order visual parameters such as spatial frequency, luminosity, and chromatic complexity. Researchers widely use and validate the IAPS as a tool for eliciting emotional responses across different cultures and age groups (Branco et al., 2023). It has proven particularly useful in studying mental disorders, including borderline personality disorder, where it has helped define specific emotional responses. The versatility and applicability of IAPS have also shown in studies addressing affective dysregulation in various mental disorders (Jayaro et al., 2008).

In the context of the above mentioned databases, it is important to consider how different types of visual stimuli, such as figurative versus abstract art, may invoke distinct emotional reactions. The semantic content or top-down information present in figurative art leads to an interpretation of what is depicted, facilitating a more direct connection with familiar experiences and emotions. Conversely, in abstract art, the information conveyed is predominantly bottom-up or low-level, i.e., it is free from the common restrictions that the visual system is used to, which might evoke a different set of emotional responses due to its open-ended nature and reliance on primary visual elements. NAPS and GAPD have also been used to stimulate and estimate emotions (Horvat, 2017). These databases have been instrumental in understanding the processing of affective pictures, with arousal consistently modulating event-related potential (ERP) component amplitude (Olofsson et al., 2008). The influence of the emotional content and physical characteristics of affective stimuli on emotional responses has been demonstrated with the emotional content being more important than the formal properties of the stimuli in evoking the emotional response (Sánchez-Navarro et al., 2006).

### 2.2 Abstract art as emotional visual stimuli

Abstract art, in contrast to figurative art (landscapes, portraits, and still lifes) frees itself from realistic representations of objects or scenes found in movements like Cubism (e.g., George Braque and Pablo Picasso) and Surrealism (e.g., Salvador Dali and Juan Miro). Painters from the New York School, such as Wassily Kandinsky, Piet Mondrian, Willem de Kooning, Jackson Pollock, and Mark Rothko, embraced an even more reductionist approach, emphasizing form, line, color, and light. This innovative approach elicits a range of raw emotional responses, liberating viewers' minds from conventional associations with color and form while fostering new connections and reactions (Kandel, 2016).

Both realistic and abstract art engage the same visual system and structures. However, the activation in case of abstract art is less specific than in case of figurative art (Kawabata and Zeki, 2004). Since abstract art doesn't represent well-defined objects—instead it is a composition of lines, spots of color, patches or simple geometric figures—it activates additional brain regions as well (Vartanian and Goel, 2004; Kawabata and Zeki, 2004). Abstract art also activates early visual processes (specialized in the perception of dots, lines, and simple objects) that are otherwise harder to access when a whole “gestalt” of a figurative image is analyzed.

Abstract art enhances the reflection of the inner stage rather than being susceptible to external visual stimuli. The individual inner state at the very specific moment of observing abstract art seems to be crucial for the insights of the viewer. As shown by Cela-Conde et al. (2013), the default mode network is activated during the later phase of aesthetic appreciation; it may be stimulated by abstract art as well.

Zhang et al. (2011) and Sartori (2014) both explored the use of low-level image features in predicting emotional responses to abstract art. Zhang's work demonstrates that these features can be used to distinguish “exciting” vs. “boring” emotions with 65% accuracy, and “relaxing” vs “irritating” emotions with 70% accuracy. Sartori's computational model can predict emotional responses as well as generate abstract paintings to elicit specific emotions. Van Paasschen et al. (2014) further support these findings, showing that observers consistently interpret the emotional content of abstract art based on its visual characteristics. In the experiment by Bashivan et al. (2019), a macaque's brain activation in the V4 area was analyzed after presenting figurative and neural network-generated pictures. The authors found that the exposition on artificial neural network-generated pictures provoked higher activation of the V4 area than figurative pictures. For this experiment, the authors used pictures generated by boosting activations of the first layers (close to the network's input) that represent simpler shapes such as curves, lines or blobs. These studies collectively highlight the potential for using low-level image features to understand and predict emotional responses to abstract art.

### 2.3 Deep neural networks

#### 2.3.1 Connections between biological and artificial neural networks

Artificial neural networks (ANN) are synthetic models of the brain's activity. In conception, they are meant to be biologically-inspired methods that leverage what we know of the brain in order to improve feature learning within machine learning. From a mathematical point of view, an ANN is a technique for function approximation. Such a function can, for example, define how to distinguish various objects from one another—dogs from cars, individual human faces or words in handwriting by different people. Since their rise in popularity, ANNs have significantly grown in scale and complexity. Our understanding of the brain has also improved. For example, the use of advanced imaging techniques such as functional magnetic resonance imaging (fMRI) and positron emission tomography (PET) has allowed us to visualize and study the brain's activity in real-time (Bandettini, 2009) and learn that, for instance, visual cortex is more active during emotions-evoking visual stimuli compared to its activity when neutral visual stimuli are presented (Gerdes, 2010).

Additionally, researchers have made great strides in mapping the brain's structure and connectivity through techniques like diffusion tensor imaging and functional connectivity analysis (Raichle, 2003). Moving forward, researchers use knowledge related to brain functions and combine machine learning with high-throughput behavioral optogenetics to stimulate very precise brain areas. They have found that the nature and magnitude of hallucinations experienced by macaques highly depend on concurrent visual input, the location of brain stimulation, and the intensity of the stimulation (Shahbazi et al., 2024).

Some would argue that current neural network architectures and their connection to the human brain are fleeting given that understanding of the dynamic processes in the brain has changed and the artificial intelligence community has understandably prioritized complex optimization-based architectures over relations to the brain (Jiang et al., 2017). On the contrary, there is much work connecting newer deep neural network architectures to a modern understanding of the brain (Marblestone et al., 2016; Richards et al., 2019). More specifically, CNNs, which are dominant and the most successful ANN models in contemporary computer vision, have their roots in the *Neocognitron* proposed by Fukushima (1980). CNNs are inspired by our understanding of the visual pathway in the brain. This pathway starts as early as in the retina, which already does a lot of visual information pre-processing (via its ganglion cells, which obtain signals from photoreceptors, as well as bipolar and amacrine cells). The information then travels through the optic nerves and chiasm (where some of the nerve fibers cross) and Lateral Geniculate Nucleus (LGN) to the ventral stream: primary visual cortex V1, visual area V2, visual area V4, and inferior temporal (IT) cortex. There are several analogies between the notion of visual cortex and CNNs, which are important from the point of view of this study:

- the layered feed-forward architectures of CNNs are inspired by a laminar organization of V1, with six identified distinct layers (Callaway, 1998; Douglas and Martin, 2004),
- the use of convolution operations in CNNs is a consequence of our notion of how simple cells in V1 perform linear filtering, by “calculating” the weighted sum of their inputs, with weights defined by the receptive field profiles (Hubel and Wiesel, 1959, 1962),
- filtering kernels in CNNs' early layers often converge (during CNN's training) to Gabor wavelets (Gabor, 1946), which were found as good models (in a least squares sense) of the receptive field profiles in simple cells in visual cortex (Daugman, 1985),
- small effective receptive fields of neurons used in early CNN layers, expanding when we move toward deeper layers, are analogous to observing the smallest receptive fields in V1 neurons (Wu et al., 2023) and extending to almost entire visual field for inferior temporal (IT) cortex neurons (Rolls et al., 2003),
- specialization of ANN neurons depends on their distance from the input image (large activations of early-layer neurons are observed for simple shapes, while deeper-layer neurons are activated more frequently for more complex objects) corresponds to a similar mechanism, neuronal tuning (Sakai and Miyashita, 1994), observed in the brain,
- heterogeneous architectures and functions of layers in ANNs (including CNNs) have their analogy in heterogeneity of anatomical properties of V1 layers; for instance in brains of primates layer 1 of V1 is almost aneuronal, while layer 4 of V1 is divided into sub-layers 4A, 4B, 4Cα, and 4Cβ (Schmolesky, 2007),
- shift property of convolution operations reasonably-well models the retinotopic mapping in V1 (and possibly in other visual cortex areas), e.g., blind spots of the retina are precisely mapped into V1, or a large portion of V1 mapped to the *fovea centralis* (known as cortical magnification) (Wandell et al., 2007).

The above list of analogies is certainly not exhaustive. They, however, support the hypothesis that CNN-based manipulations of visual inputs may offer a reasonable control of the evoked reactions in human's brain, and perhaps better-localized than the one evoked by images selected from a larger pool of naturalistic photographs or synthesized by most recent and popular generative AI language-prompted models.

For a given input image, areas or features which give the model stimulus can also be calculated. Using this information, architectures can be built to represent how visual cortices process stimuli and the signals that cause neurons to fire. Much like neural networks, our brain depends on nonlinear transformations and pooling of signals in order to perform complex tasks, such as recognition. Yamins et al. (2014) demonstrate how CNNs can be optimized to perform object recognition tasks like humans, by building layers that represent visual cortices in sequences that mimic the interconnected regions of our brain.

#### 2.3.2 “Dreamception”

Originally built in 2014 to beat the state-of-the-art classification performance on the ImageNet Large-Scale Visual Recognition Challenge (ILSVRC14), the Inception network is a deep (originally 22-layer) CNN that processes images differently than previously-proposed CNNs (Szegedy et al., 2015). The Inception model introduced two custom modules called *inception modules*, which combine several convolution layers and a pooling layer with a filter concatenation for the purpose of processing visual information at multiple scales during feature extraction. By stacking convolution layers with different sized kernels, the model can decompose spatial features and create more abstract representations of the data, which improved upon the best classification scores in the ILSVRC14 as a result. Supplementary Section 1 contains a visualization of a more recent Inception network (composed of 144 layers) and its inception module used in this work.

In 2015, Google researchers demonstrated that the same classification tool could be used in a feedback loop to emphasize what the network believes it is currently seeing (Mordvintsev et al., 2015b). As a result, “Inceptionism,” which allows for a qualitative assessment of what the neural network is “seeing” at each respective layer, was created. The Inception model, starting from any image (e.g., a white noise), was used in an optimization loop, in which activations of selected ANN's section were boosted by iterative alterations of the input. This procedure creates vivid, hallucinogenic images allowing researchers to see what features in the input images the network is sensitive to, for either each single neuron, separate feature maps, entire layers or arbitrary sets of neurons.

#### 2.3.3 Deep learning-based generative models

The quest for building models that “understand” visual inputs has existed since the birth of artificial intelligence in the 1960's. In principle, generative models first learn the distribution of the training data representing a given domain, and then are able to sample from that distribution and generate new domain-specific exemplars.

**Figure 3:**
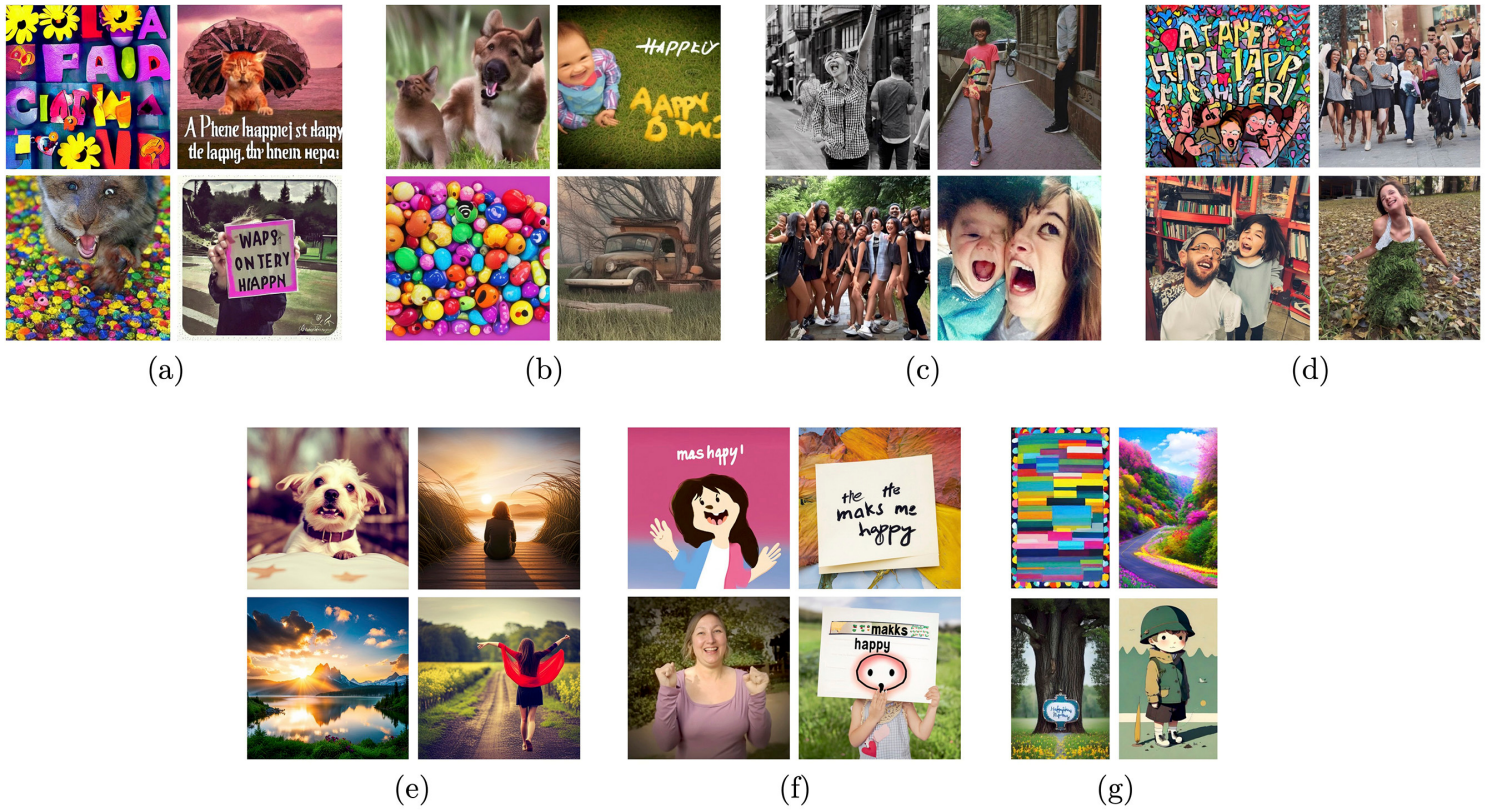
Sample images generated by a few examples of modern text-to-image generative models for the text prompt “A picture that makes me happy”: Stable Diffusion v1.4 **(A)**, Stable Diffusion v1.5 **(B)**, Stable Diffusion v2.1-base **(C)**, Stable Diffusion v2.1 **(D)**, DreamStudio AI **(E)**, Adobe FireFly **(F)**, and Wombo **(G)**.

**Figure 4:**
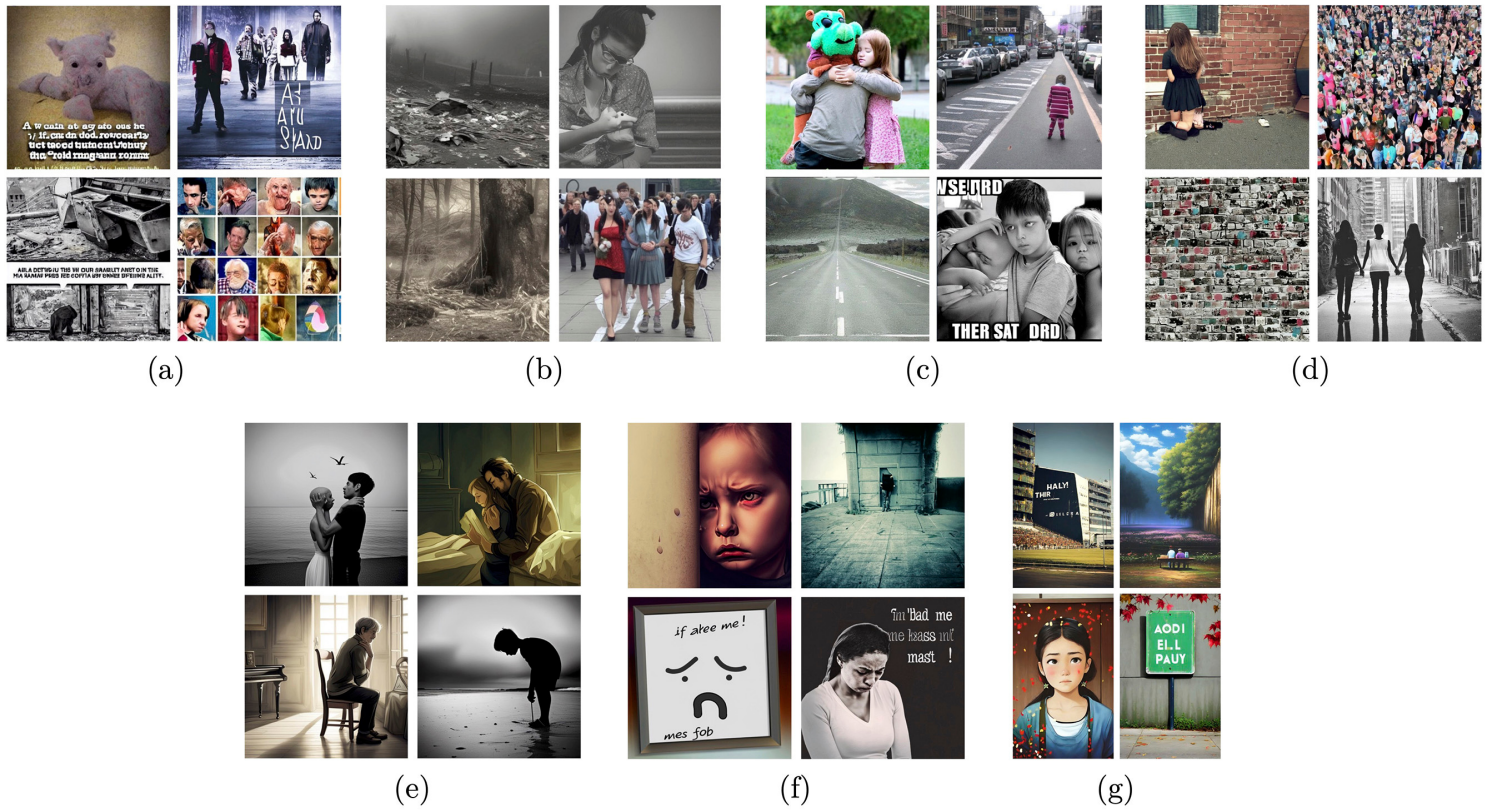
Sample images generated by a few examples of modern text-to-image generative models for the text prompt “A picture that makes me sad”: Stable Diffusion v1.4 **(A)**, Stable Diffusion v1.5 **(B)**, Stable Diffusion v2.1-base **(C)**, Stable Diffusion v2.1 **(D)**, DreamStudio AI **(E)**, Adobe FireFly **(F)**, and Wombo **(G)**.

Modern deep learning-based generative models create representations in their latent spaces, which then are used to revert the encoding process and generate new samples, hopefully following the distribution of the original training data, although not simply duplicating the training data. The structure of the latent space is the biggest mystery of such models, and may be shaped only partially. For example, Variational Autoencoders (Kingma and Welling, 2019), Adversarial Autoencoders (Makhzani et al., 2016) or StyleGAN (Karras et al., 2020b,a, 2021) models implement mechanisms to organize latent space representations of input data samples in a way to disentangle various factors of data variation, give them semantic meaning and make these factors—to the maximum extent possible—statistically independent. Sampling in the latent space can be also controlled by additional information such as encoded language prompts. These hybrid text-to-image generative models, if trained collectively in an end-to-end manner, are hoped to learn semantically-valid connections between language descriptions and visual outputs (images and videos). And while this hope is partially satisfied, such that these models are able to generate images that exhibit some semantic structure, the accuracy of the alignment of the generated samples with actual human perception is unknown, and when examined on a case-by-case basis, it is often disappointing. Figures 3, 4 show examples of synthetic images generated by several state-of-the-art text-to-image generative models, for two prompts: “A picture that makes me happy” and “A picture that makes me sad,” respectively. The first observation is that these models “understand” the prompts on a higher semantic level, offering people and texts combined with colorful or gray-toned objects as the results, as those exemplars probably prevailed in training datasets. The second observation is that human perception of these image generations would be widely different than the prompts would suggest. For example, we can look at deformed bodies and faces, even in samples that are supposed to make us happy, which *de facto* actually evoke disgust or fear. So while the existence of such models is noted, and hopes related to correct generations are present, they are not yet at a development stage which would allow their immediate application for controlled stimulation of emotions.

**Our work differs from the past works mentioned above** in that we examine a different generative framework that synthesizes visual stimuli at a lower semantic level. The motivation, and our hypothesis, is that stimuli that are semantically less complex yet carefully synthesized, result in images affecting brain functional regions that are possible to be identified with a greater precision compared to localizing areas storing and processing semantically-meaningful information (Binder et al., 2009). If that hypothesis is true, we would be able to build more accurate models of emotional reactions and thus create affordable and widely-accessible tools that could help people control their emotions.

### 2.4 The LockLuck tool

*LockLuck–More than Cards*™is a tool created in 2015 to increase self-esteem, self-value and self-confidence in life and business coaching^1^. The goal for this tool was to facilitate as well as to encourage a deep coaching conversation. The tool is composed of three decks of cards (30 questions, 84 graphics, and 56 quotes), instructions and a guide. The questions guide the client in approaching, recognizing, dominating, and negotiating with the inner judgmental voice.

**Figure 5:**
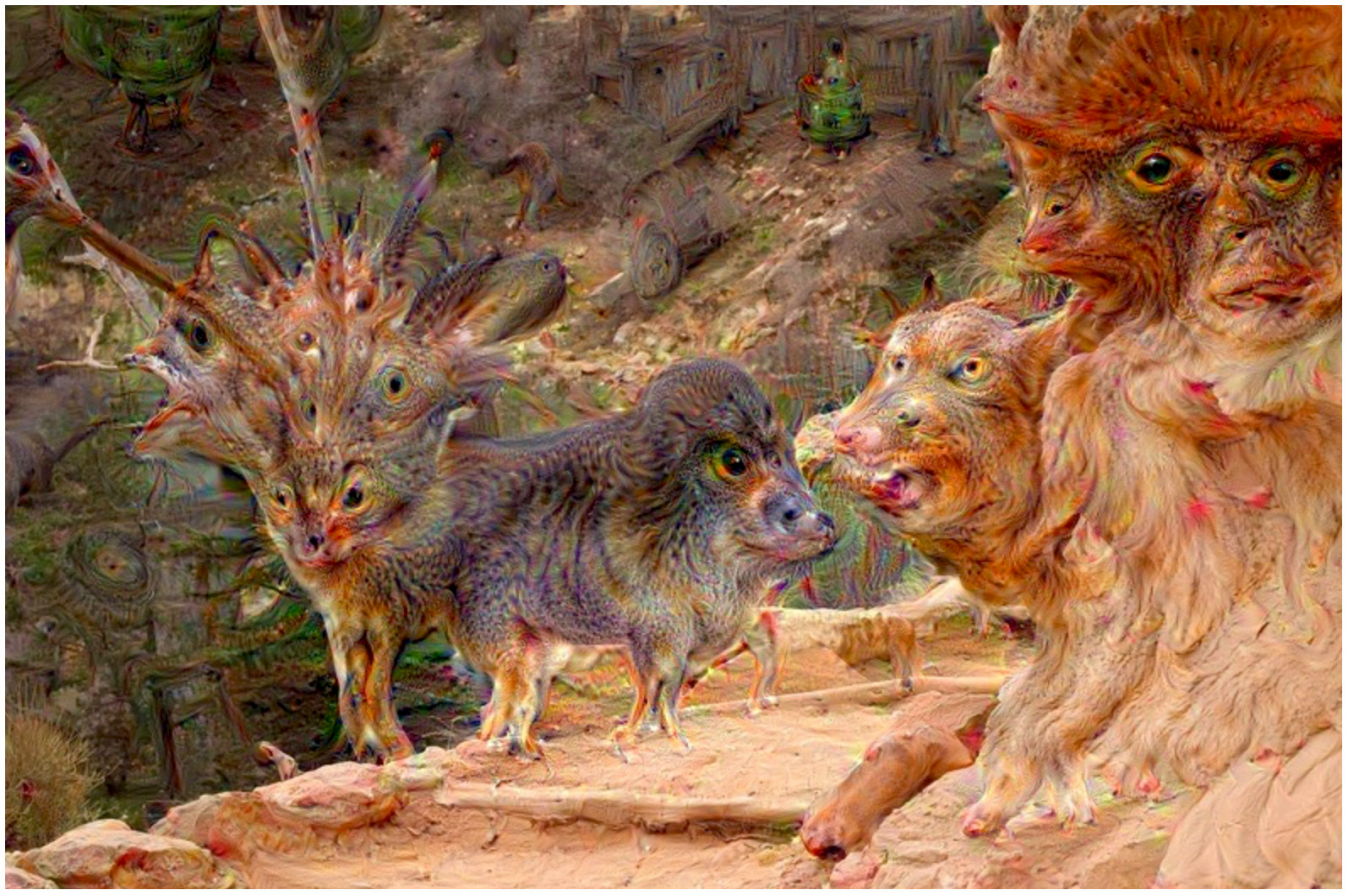
An example of the “deep-dreamed” image synthesized for the *LockLuck–More than Cards* tool. Instead of a noise, the starting point was a natural image of a mountain trail.

After answering a question, the cards with graphics and quotes play their role as linguistic and visual metaphors. In particular, the “multidimensional” nature of the graphics (e.g., natural pictures transformed by neural networks to overlay the “deep dream” layer, as illustrated in Figure 5) helps in building metaphors used to express what the clients experience right now. That is, when the client talks about the picture, it makes it easier to reach the hidden information (to “unlock” it) that was not immediately available after asking the question. The quote cards inspire, break an impasse, deliver additional information, or deepen the insights that flow from the graphics cards. The quotes and questions are not used in the research presented in this report. The *LockLuck–More than Cards*™tool is, to our knowledge, the first tool using neural network-transformed pictures applied to coaching processes.

## 3 Materials and methods

### 3.1 Synthetic image generation

Synthetic images used in this study were generated by first constructing a uniform gray 800 × 800 pixel base image. Then Gaussian noise (with a mean value 0 and a standard deviation 12.75) was generated from a fixed random seed and added to the image. Subsequently, the image was updated in an iterative manner in order to maximize the activations of a given layer or sub-layer. In each iteration of the process, the image would be passed to the Inception model as input, and the activations of the target layer would be recorded. Then, by calculating the gradients of (the sum of) the activations with respect to the input image and applying them to the image, gradient ascent was used to update the image in a manner that maximally increased the total of the given activations. During this step (i.e., gradient ascent), updates were applied with a fixed step size of two. Following this, pixels within the updated image were restricted to be integers within the range of [0,255] (e.g., valid pixel values) using clipping and rounding. Finally, the updated image would then be fed back to the model and used as the starting point in the next loop. This process was repeated until the image no longer changed between loops as this indicated that the generated image maximized the target activations in an (at least locally) optimal manner.

Following this protocol, images were generated for all 144 layer and sub-layer within an Inception model that had been trained on ImageNet. In order to ensure that the experiments measured the characteristics of each layer/sub-layer rather than the characteristics of individual ideal images, multiple images were generated and evaluated for every layer/sub-layer. Following the observation that every image generated from a given layer/sub-layer ended up being highly visually similar to the other images generated from the same layer/sub-layer, it was determined that five images per layer/sub-layer would be enough to assess the characteristics of each layer/sub-layer. This led to the generation of a total of 720 images that were used in the experiment reported in this study.

### 3.2 Scales

Participants viewed the synthesized images (as described in Section 3.1) and responded to them using sliders to rate them on two scales: valence and arousal. The sliders output values were in the range of 0 to 100. The scales were visually represented using five self-assessment manikin figures (SAM) (Bradley and Lang, 1994), each accompanied by labels for the endpoints, as shown in Figure 2. For valence, the scale ranged from a sad face to a smiling face, labeled “unhappy” and “happy,” respectively. Arousal was depicted using figures ranging from calm and relaxed to excited and interested, with endpoints labeled “calm” and “excited,” respectively. This method of ratings along these dimensional scales is very common among emotion-eliciting images such as for the ISEE (Kim et al., 2018) and IAPS (Lang et al., 2005).

### 3.3 Participants

The initial sample included 153 participants, however, 3 of the participants did not complete the study and their data was removed. Thus, our analysis was based on 150 participants (98 female) with mean age = 26.16 years, S.D. = 9.137. The participants for this paper were recruited on-line using Prolific^2^. Each participant was compensated monetarily for their time. The experimental procedure received approval from the Institutional Review Board (IRB) at the University of Notre Dame, IN, USA. The experiment was conducted strictly in accordance with the approved guidelines of the committee.

### 3.4 Acquisition protocol

Participants completed a Qualtrics survey (Qualtrics, 2024) consisting of a consent form, several demographic items, mood and self-esteem items, and then the items to rate the synthetic images on scales of arousal and valence as described in Section 3.2. In order to reduce fatigue, all participants were not exposed to all 720 generated images. Rather, the 144 layers were divided into three groups of 48 layers each. Participants were randomly assigned to one of these groups of layers. For each of these 48 layers, a participant rated one of the 5 generated images for that layer, chosen randomly. Thus, each participant rated a different set of 48 images in random order. Once the 48 images were rated, the participants were thanked and given a survey code to receive their Prolific credit. Participants were instructed during consent to withdraw at any time by closing their browser should they no longer wish to continue with the experiment.

## 4 Results

### 4.1 Image visual properties

Each layer in the model was applied to one of five randomly generated Gaussian noise images to yield five stimulus images per layer. Participants were presented with a random sample of one of these five images. Supplementary Tables S1–S3 present the mean arousal and valence rating across participants for each of the model layers as well as six image metrics: Hue, Saturation, Brightness, JPEG compression percentage, and two measures of visual clutter: Feature Congestion and Sub-band Entropy (Rosenholtz et al., 2007).

Hue, Saturation, and Brightness were computed by utilizing the cvtColor method of the Python OpenCV library (Bradski, 2000) to convert the image to the HSV (Hue, Saturation, and Value) colorspace (where Value is semantically the same as Brightness) and then calculating the mean of each attribute rounded to three decimal places. It is important to note, however, that we did not have a control over individual monitor settings and the assumption about uniform color and contrast settings across subjects is made in this case.

The JPEG compression percentage was calculated by a custom Python program that computed the percentage of jpeg compression with quality set to 80 as compared to the original idealized file size (800 × 800 × 3 bytes) using the Pillow image processing python library (Clark, 2015) for jpeg compression and the Python os library to obtain the reduced file size. Feature Congestion and Sub-band Entropy were computed using the Matlab library offered by Rosenholtz et al. (2007).

**Figure 6:**
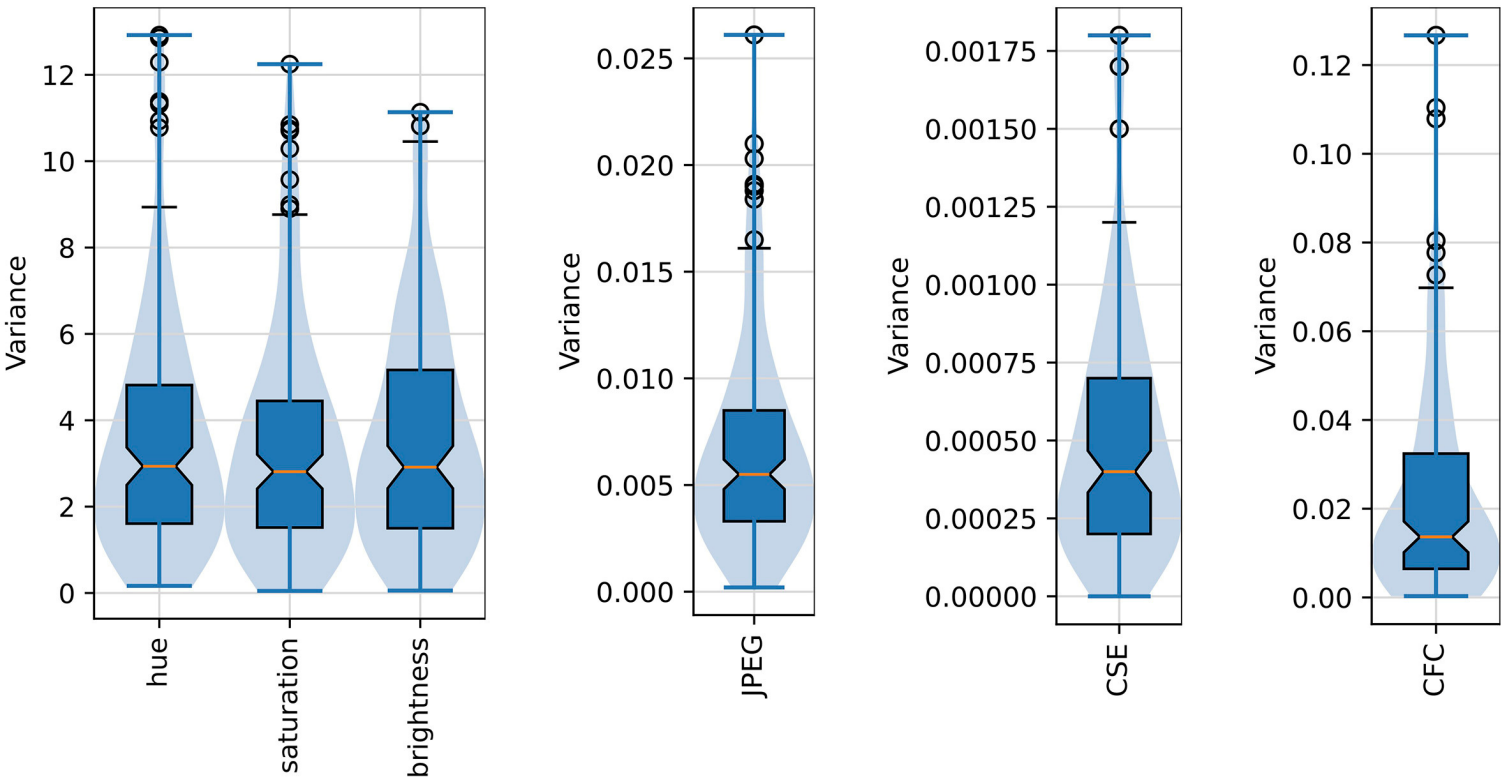
Distribution of variances of the six selected image properties of the “deep dreamed” images used in this study. Median values are marked as red middle bars, height of each boxes corresponds to an inter-quartile range (IQR) spanning from the first (Q1) to the third (Q3) quartile, whiskers (marked by short black horizontal bars) span from Q1–1.5*IQR to Q3+1.5*IQR, outliers are shown as black circles, and longer horizontal blue bars denote minimum and maximum values. Notches in the boxplots represent 95% confidence intervals of the median. The violin plots are overlaid to provide a better visualization of the actual distribution shapes.

Figure 6 shows the distribution of variances of the six image properties described above. The variances of all six properties are low, what suggests that images generated for each layer, and originating from different noise seeds, are characterized by very similar global image properties.

### 4.2 Correlation with affective judgments and image visual properties

**Figure 7:**
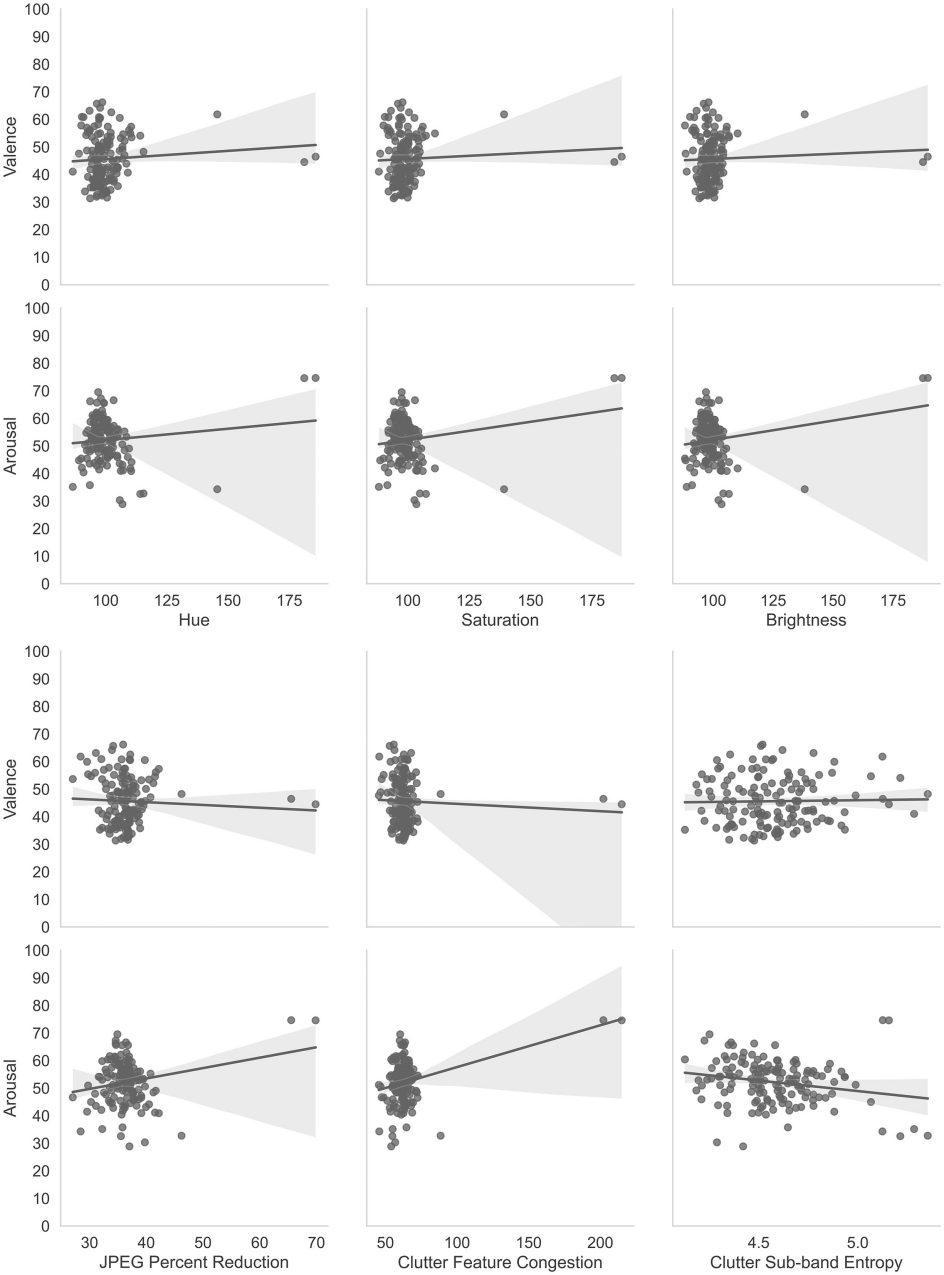
Correlations of affective judgments with visual properties of images. The two most obvious outliers in each of the distributions are for the same layers, namely. convd0 and maxpool0. The layer, cond0_pre_relu was also an outlier for Hue, Saturation, and Brightness.

Figure 7 shows scatter plots and best-fit lines for arousal and valence judgments as compared with the 6 image properties described in Section 4.1.

Multiple linear regressions were performed using IBM SPSS Statistics software (IBM, 2023) to predict arousal and valence judgments from the image characteristics (Hue, Saturation, Brightness, JPEG Percent Compression (JPEG), Clutter Feature Congestion (CFC) and Clutter Sub-band Entropy (CSE)). These two multiple linear regressions resulted in a statistically significant model for arousal, *F*_(6, 137)_ = 13.393, *p* < 0.001, R2 = 0.370. Thus, the arousal model explains or predicts 37% of the relationship between the dependent and independent variables. The individual predictors for arousal were examined further and indicated that Hue (t = –2.562, *p* < 0.05), CFC (t = 2.608, *p* < 0.01), and CSE (t = –3.946, *p* < 0.001) were significant predictors but saturation (t = –0.587, *p* = 0.558), brightness (t = 1.769, *p* = 0.079), and JPEG compression (t = –0.755, *p* = 0.451) were not. The model for valence was not statistically significant, *F*_(6, 137)_ = 1.814, *p* = 0.101, R2 = 0.033. Thus, the valence model only explains or predicts 3.3% of the relationship between the dependent and independent variables. The individual predictors for valence were examined further and indicated that none were statistically significant predictors: Hue (t = 1.044, *p* = 0.298), Saturation (t = 0.741, *p* = 0.298), Brightness (t = –1.172, *p* = 0.243), JPEG compression (t =-1.304, *p* = 0.195), CFC (t = –0.129, *p* = 0.897), and CSE (t = –0.281, *p* = 0.779). These results are consistent with previous studies that have shown an arousal-complexity bias (Madan et al., 2018) where arousal was affected by image complexity as measured by Clutter Feature Congestion and Clutter Sub-band Entropy. Madan et al. (2018) also found that JPEG Compression did not affect arousal or valence measures which we also demonstrated. Thus, these analyses are complementary to the main experiment to show that basic image properties are not significant predictors of arousal and valence with the exception of image complexity and hue (Kuzinas et al., 2016), which aligns with previous observations reported in the literature.

Pearson correlations from the multiple linear regression analyses are presented in Table 1.

**Table 1:** Pearson correlations of arousal and valence judgments with independent variables of image characteristics.

	**Arousal**	**Valence**	**Hue**	**Saturation**	**Brightness**	**JPEG**	**CFC**
Hue	0.120	0.080					
Saturation	0.187^*^	0.060	0.990^***^				
Brightness	0.204^**^	0.050	0.986^***^	0.999^***^			
JPEG	0.215^**^	–0.053	0.807^***^	0.805^***^	0.808^***^		
CFC	0.343^***^	–0.056	0.814^***^	0.848^***^	0.858^***^	0.874^***^	
CSE	–0.216^**^	0.023	0.364^***^	0.343^***^	0.344^***^	0.236^**^	0.340^***^

(Significance levels: ^*^ *p* < 0.05, ^**^p < 0.01, ^***^ *p* < 0.001).

### 4.3 Association of affective judgments with mood

Two items were presented in the pre-questionnaire regarding the participant's mood on the day of their participation. The participant was prompted to rate their attitude with the item, “My attitude today is ...” with the following three mutually-exclusive responses provided: “Positive,” “Neutral,” or “Negative.” Participants were also asked a question about their self-esteem with the prompt, “I have high self-esteem” and then given five mutually-exclusive responses to choose from: “Definitely true,” “Probably true,” “Neither true nor false,” “Probably false,” and “Definitely false.” The responses to these items were compared with a participants' mean arousal and mean valence scores computed across all images that the participant was presented in order to determine if attitude or self-esteem affected their overall arousal and valence judgments with significance judged as *p* < 0.05. A one-way ANOVA revealed that there was not a statistically significant difference in mean valence for attitude ratings [*F*_(2, 147)_ = 2.44, *p* = 0.091]. Similarly, a one-way ANOVA revealed that there was not a statistically significant difference in mean arousal for attitude ratings [*F*_(2, 147)_ = 2.41, *p* = 0.786]. Self-esteem ratings also did not appear to show any association with valence and arousal judgments. A one-way ANOVA revealed that there was not a statistically significant difference in mean valence for self-esteem ratings [*F*_(4, 145)_ = 1.45, *p* = 0.221]. Also, a one-way ANOVA revealed that there was not a statistically significant difference in mean arousal for self-esteem ratings [*F*_(4, 145)_ = 1.58, *p* = 0.183].

**Figure 8:**
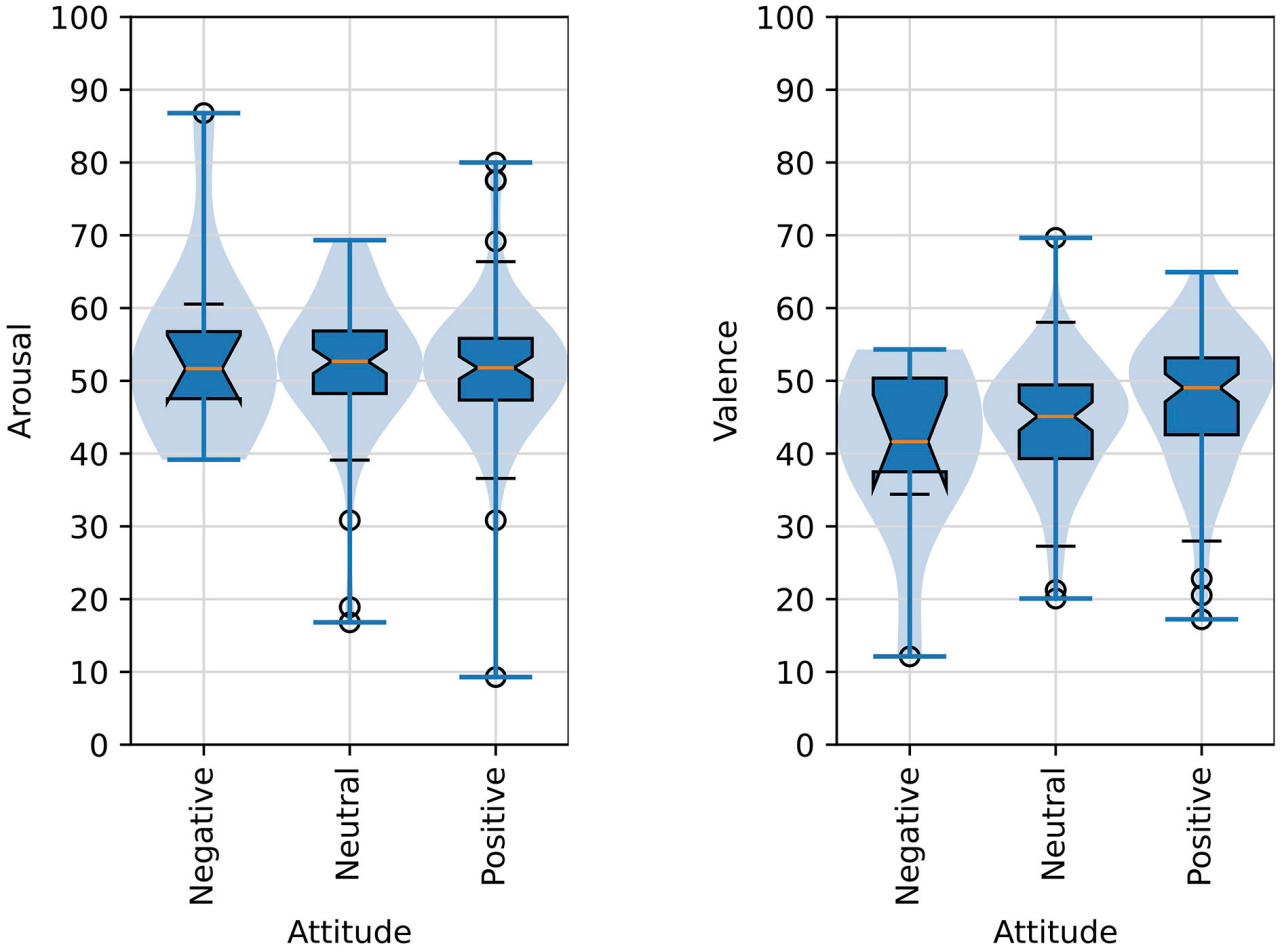
Distributions of arousal and valence scores as function of the affective judgments of images and attitude of participants. The formatting of boxplots and violin plots is the same as in Figure 6.

Figure 8 shows violin plots and overlaid box plots for arousal and valence judgments as compared with participants' attitude.

**Figure 9:**
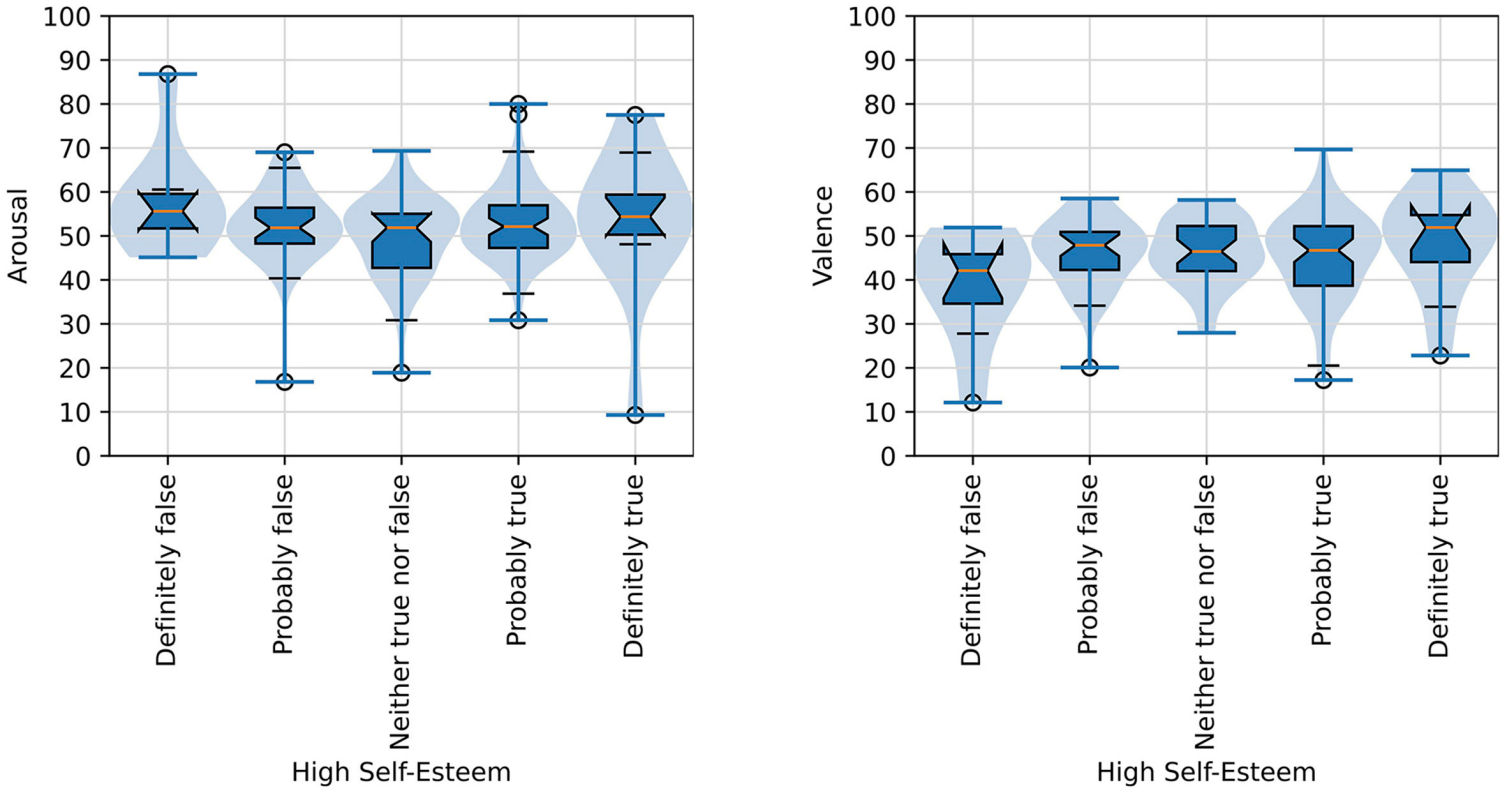
Distributions of arousal and valence scores as functions of the affective judgments of images and high self-esteem rating of participants. The formatting of boxplots and violin plots is the same as in Figure 6.

Figure 9 shows violin plots and overlaid box plots for arousal and valence judgments as compared with participants' ratings of high self-esteem.

### 4.4 Self-reported arousal and valence analysis

As illustrated in Section 3.1, the synthesized images that increase averaged activations of all neurons located in single layers have visually different appearance depending on that layer. This Section presents an analysis in which we verify the hypothesis that such images evoke different reactions (measured as self-reported arousal and valence) depending on the CNN layer selected to boost that layer's neuron activations through input image perturbations.

#### 4.4.1 Independent analysis of valence and arousal scores

**Figure 10:**
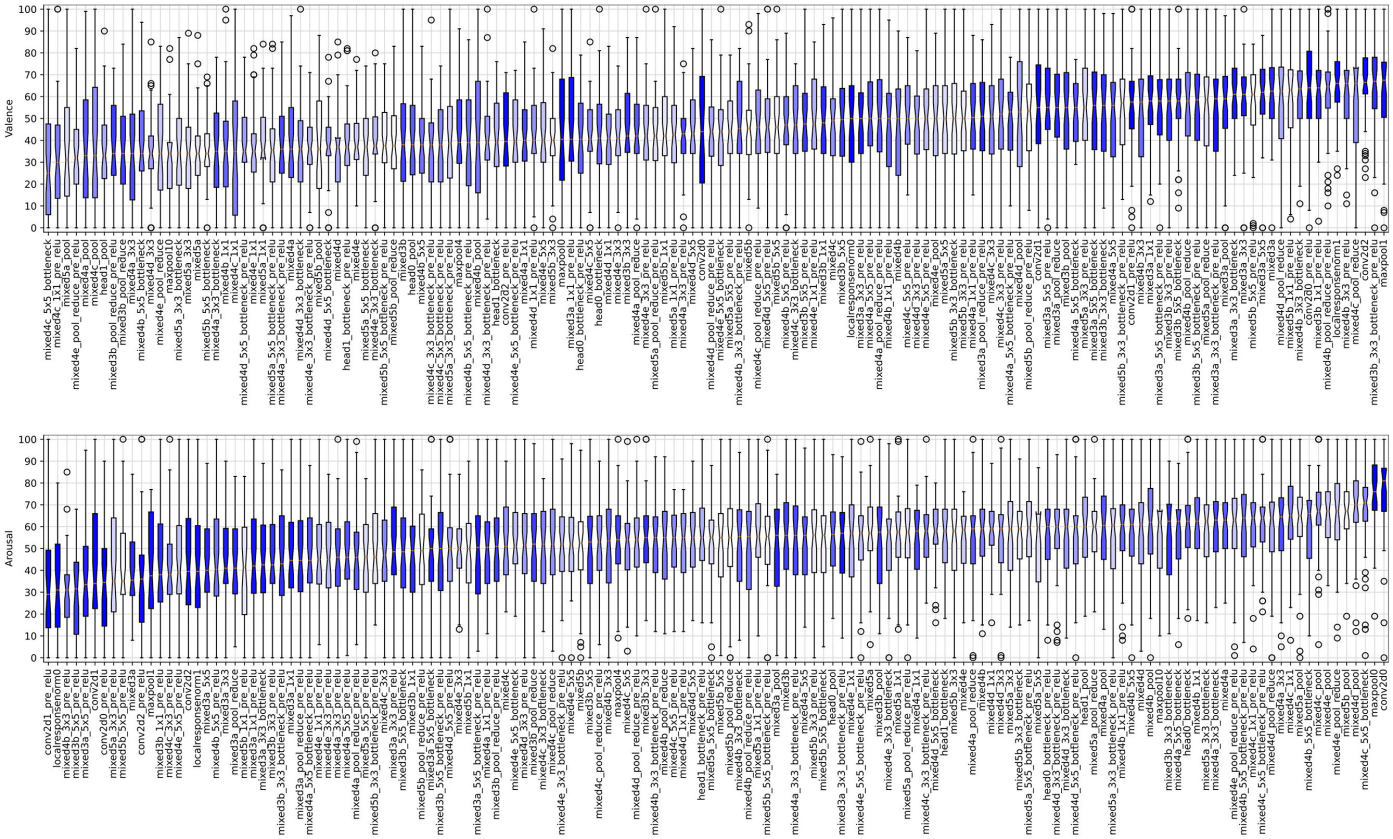
Boxplots illustrating the valence (top plot) and arousal (bottom plot) scores sorted by the median values obtained for images boosting activations in layers listed on the horizontal axis. The formatting of boxplots is the same as in Figure 6, except for the color coding: boxplots representing results obtained from deeper layers are light-shaded, while those representing results obtained from the layers closer to the input are darker. The versions of these plots for scores sorted by the layer's position in the Inception network are provided in Supplementary Figure S13.

Figure 10 shows boxplots summarizing the valence and arousal, respectively, self-reported by participants looking at images that maximize neuron activations of a single layer shown on the X axis. Since the sliders output values of the response items were in the range of 0 to 100, the value 50 is considered as a neutral reaction, values closer to 100 indicate high arousal/valence reaction, and values close to 0 correspond to low arousal/valence reaction. The plots are sorted by the position of the layer in the CNN: from the first convolutional layer (conv2d0_pre_relu; see Supplementary Figure S1 for the full topology of the Inception-V3 neural network used in this study) looking at the image, until the last convolutional layer (mixed5b) feeding the fully-connected output (performing linear classification) layer. There are a few interesting observations (O1-O3) we can make:

**O1:** There are layers for which images maximizing their activations also evoke non-neutral reactions of participants looking at these images.

For instance, statistically significant higher/lower than neutral arousal can be noticed for conv2d0/mixed3d_5x5_pre_relu, and statistically significant higher/lower than neutral valence can be observed for mixed4d_pool_reduce_pre_relu/mixed5a layers.

**O2:** There are layers for which images maximizing their activations do not translate to non-neutral arousal or valence reactions.

For instance, localresponsenorm0 and mixed3b_3x3_bottleneck_pre_relu are distributed around the neutral score (50) of the self-reported valence and arousal, respectively. It is also interesting to see that such neutral responses can be observed for images boosting activations of layers located in various places in the network, which is a segue to our last observation:

**O3:** The self-reported average valence and arousal values do not seem to depend (e.g., monotonically) on the layer's position within the network. Indeed, there are *types* of layers, whose outputs—when boosted—create images that result in statistically significant differences in participants' self-reported reactions, both related to valence and arousal.

**Figure 11:**
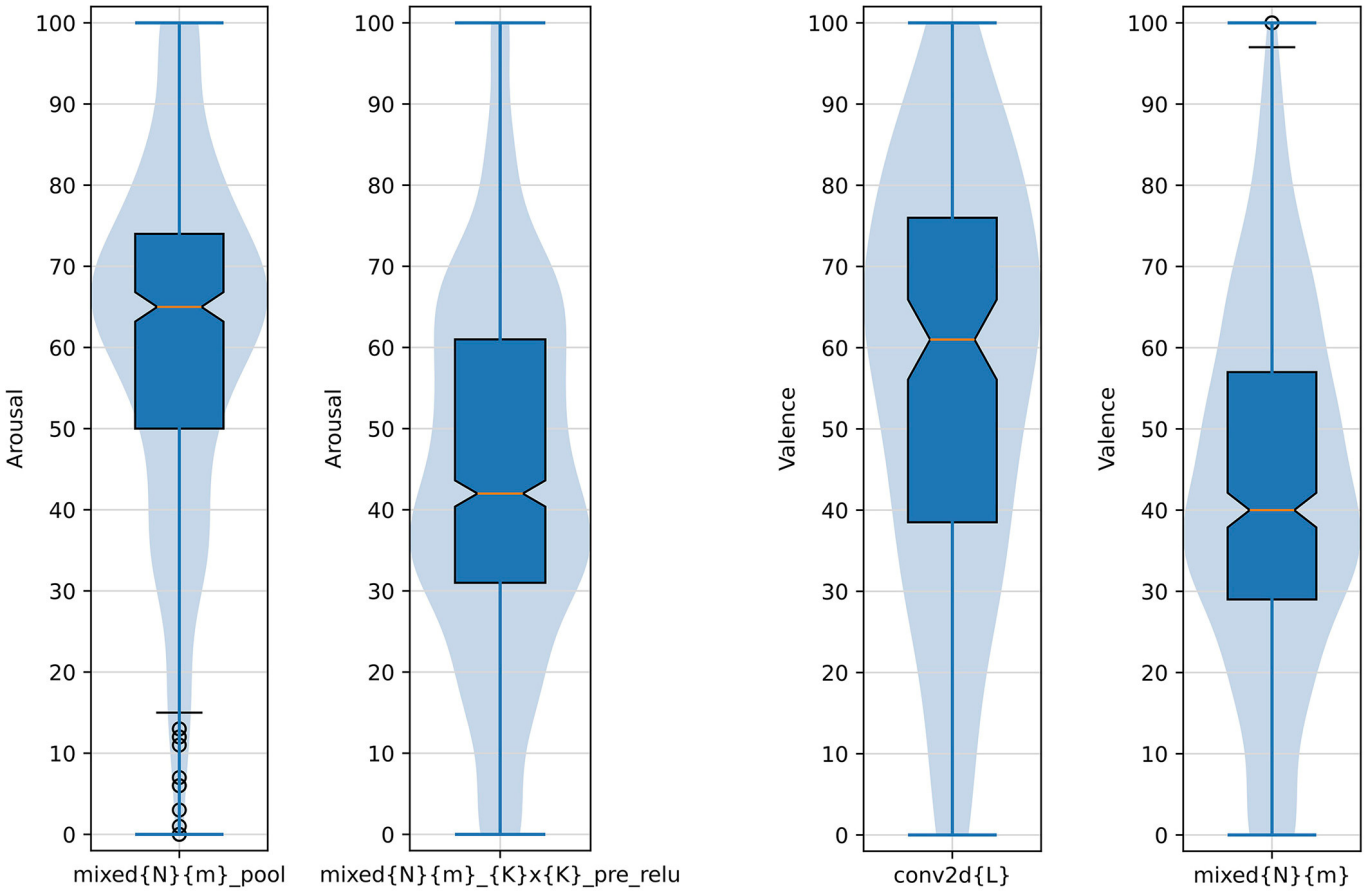
Arousal and valence scores aggregated over example *types* of layers resulting in statistically significantly different responses between such types. Numbers and letters defining the groupings are: *N*∈{3, 4, 5}, *K*∈{3, 5}, *L*∈{0, 1, 2}, and *m*∈{*a, b, c, d, e*}. The formatting of boxplots and violin plots is the same as in Figure 6.

Indeed, Figure 10 does not reveal clear linear trends in average valence and arousal as a function of the layer's depth. However, when arousal and valence responses are grouped by choosing a layer type (e.g., direct outputs of the inception modules), we see statistically significant differences in average valence and arousal responses aggregated over the selected layers. In particular, as shown in Figure 11 on the left two plots, grouping images boosting activations of layers mixed{N}{m}_pool (*N*∈{3, 4, 5} and *m*∈{*a, b, c, d, e*}) result collectively in statistically significantly higher arousal scores than for images obtained for layers mixed{N}{m}_{K}x{K}_pre_relu (*K*∈{3, 5}). Similarly, as see in Figure 11 on the right two plots, grouping images boosting activations of layers conv2d{L} result collectively in statistically significantly higher valence scores than for images obtained for layers mixed{N}{m}.

#### 4.4.2 Analysis of joint distributions of valence and arousal scores

In the previous section we made the observation that both valence and arousal are individually dependent on the image type. In this section we analyze both responses jointly.

**Figure 12:**
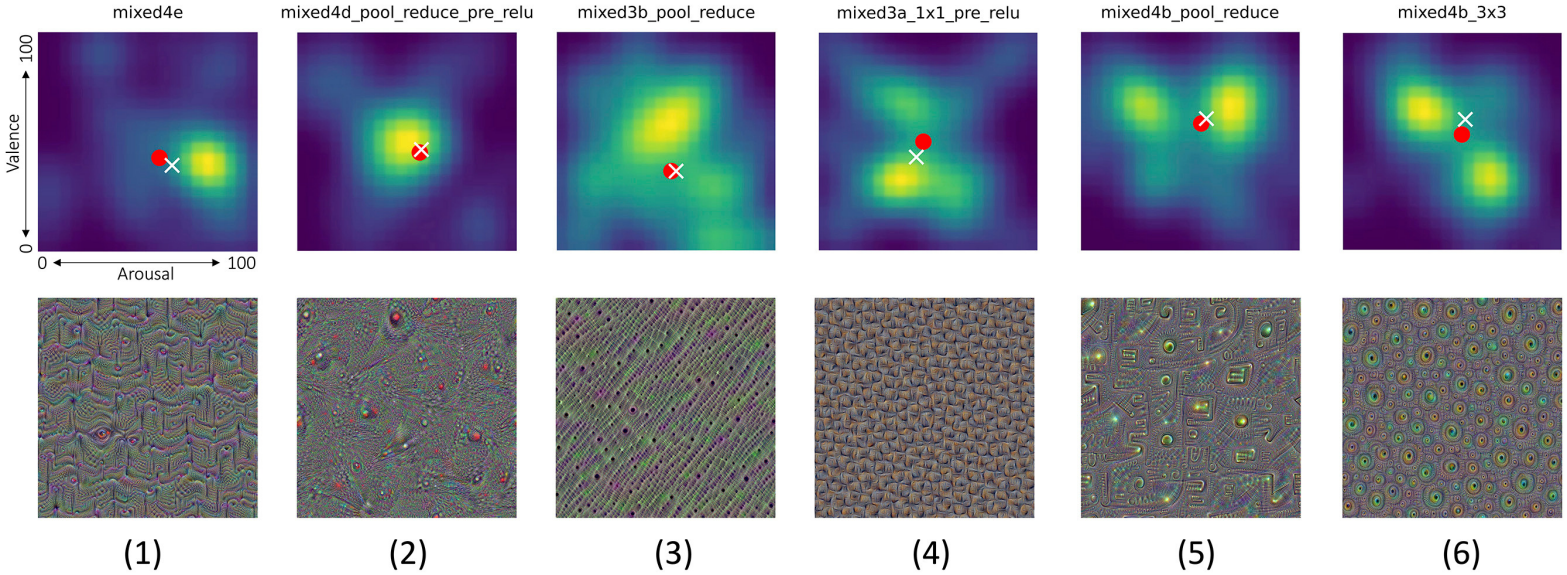
**Top row** shows joint distributions of valence (vertical axis) and arousal (horizontal axis) responses from all participants (smoothed to create heat maps for better visibility) to the corresponding pictures invoking these distributions shown in **bottom row**. Six interesting cases are visualized, from left to right: (1) the lowest Shanon's entropy of the valence/arousal joint score distribution, (2) the mean of entropy, as in (1), and the distance of the average point from the neutral arousal and valence (50,50), (3) opposite to (1): the largest Shanon's entropy of the valence/arousal joint score distribution, (4) the case in which the arousal score is spread across participants, but the valence has a bimodal distribution, (5) opposite to (4): the valence score is spread across participants, but the arousal has a bimodal distribution, and (6) a bimodal distribution of scores with one mode centered in low arousal and high valence, and the second mode centered around high arousal and low valence. White cross and red circle markers represent the mean and median values of all responses, respectively. Joint distributions corresponding to all 144 Inception v3 layers are shown in Supplementary Figures S2–S4. Additionally, pictures generated for Inception v3 layers which obtained various combinations of extreme median arousal and valence values are shown in Supplementary Figures S5–S12.

For each synthetically-generated image, boosting a single CNN layer, we can calculate and visualize a joint probability distribution of valence and arousal self-reported by the participants. These distributions reveal often complicated relations between both reactions, namely:

(a) There are layers which are boosted by images causing non-neutral and *very repeatable* reactions across all participants. Such layers can be identified by picking the joint probability density plot with the minimum entropy (across all joint distributions), as shown in Figure 12-1. The entropy-based measure applied in this case is Shannon's entropy calculated for values of the joint probability density plots (represented by pixel intensities in Figure 12), as proposed by Crum et al. (2024) to measure the entropy of CNN models' saliency maps.
(b) There are layers for which generated images do not evoke strong reactions, as shown in Figure 12-2.
(c) Opposite to (a), there are layers for which generated images evoke highly diverse reactions, as shown in Figure 12-3. Such layers can be identified by searching for maximum Shannon's entropy of the joint distribution plots.
(d) A few layers end up with synthetic images that result in a bimodal distribution for only one type of the responses, while producing very diverse responses in the other type. One example is shown in Figure 12-4, which demonstrate how images boosting mixed3a_1x1_pre_relu layer ended up with a bimodal distribution of the valence scores, while generating more uniform responses along the arousal dimension. Figure 12-5 shows an opposite case, when arousal scores are more polarized than the valence scores.
(e) Finally, very interestingly, there are layers, which—when boosted–synthesize images that generate responses polarized in both dimensions, as shown in Figure 12-6: there are apparently two clear groups of participants who either self-reported low arousal along with high valence, or high arousal along with low valence.

**Figure 13:**
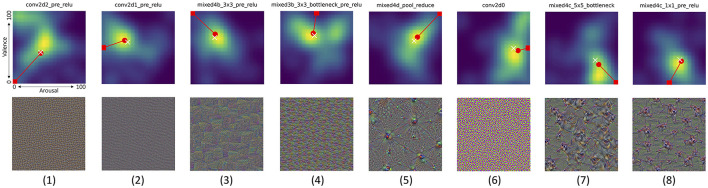
Joint distributions of valence/arousal scores, as in Figure 12 for another eight interesting cases (columns), in which the average arousal/valence scores obtained from the participants when looking at pictures (shown in the bottom row) were the closest to the eight selected combinations of the arousal and valence scores (marked by the red rectangles). From left to right: (1) negative arousal and valence, (2) negative arousal and neutral valence, (3) negative arousal and positive valence, (4) neutral arousal and positive valence, (5) positive arousal and valence, (6) positive arousal and neutral valence, (7) positive arousal and negative valence, and (8) neutral arousal and negative valence. The center (50,50) of each plot corresponds to neutral arousal and valence reactions. White cross and red circle markers represent the mean and median values of all responses, respectively. Joint distributions corresponding to all 144 Inception v3 layers are shown in Supplementary Figures S2–S4. Additionally, pictures generated for Inception v3 layers which obtained various combinations of extreme median arousal and valence values are shown in Supplementary Figures S5–S12.

Additionally, Figure 13 illustrates example layers, for which median arousal and valence scores were closest to (a) one of the four corners of the arousal-valence plane (minimum arousal and maximum valence simultaneously, or the opposite: maximum arousal and minimum valence simultaneously, or both arousal and valence either minimum or maximum at the same time), or (b) one of the sides of the arousal-valence plane (meaning that only one response type was strong, while the second was neutral).

The main conclusion from the above observations, presented in both subsections, is that a simple mechanism of iterative image perturbation, with a goal to increase average activation of artificial neurons located in a single layer of the CNN, allows to synthesize images that evoke non-neutral reactions measured by arousal and valence. Some of these images evoke reactions that are highly repeatable across participants, and some other images evoke polarized reactions. These results support the assumption that images that are stimulating artificial neural structures resembling functions of the visual cortex, yet doing this at a lower, in a sense, semantic content level, may serve as a mechanism of evoking reactions similar to visual stimuli that are interpretable to humans, such as those presented in Section 2.3.3.

## 5 Discussion

Our analysis of images synthesized to maximize neuron activations in different CNN layers revealed varying self-reported arousal and valence responses from subjects. Certain layers, such as conv2d0 and mixed3d_5x5_pre_relu, produced images that led to significantly higher or lower arousal levels, while layers like mixed4d_pool_reduce_pre_relu and mixed5a evoked strong valence reactions. Interestingly, some layers, including localresponsenorm0 and mixed3b_3x3_bottleneck_pre_relu, resulted in neutral responses, highlighting that not all activations translate to significant emotional reactions.

The lack of a clear monotonic relationship between the depth of the layer and the emotional response underscores the complexity of neural representations in influencing affective judgments. Notably, when responses are grouped by layer types, such as inception module outputs, statistically significant differences in arousal and valence are observed, indicating that the type of layer, rather than its position, plays a crucial role in shaping emotional responses. Additionally, the examples of bimodal distributions of scores, with one mode centered in low arousal and high valence, and the second mode centered around high arousal and low valence, suggest that individual differences in creating judgments and affecting emotional reactions may play a role.

Our study offers several key insights into the relationship between visual stimuli characteristics, initial mood states, and the resulting affective judgments of arousal and valence. The regression analysis identified hue, feature congestion, and sub-band entropy as significant predictors of arousal, explaining 37 percent of the variance. This finding supports the arousal-complexity bias observed in prior research, indicating that more complex visual stimuli tend to elicit higher arousal levels. Conversely, the valence model did not yield significant predictors and explained only 3.3 percent of the variance, suggesting that the pleasantness or unpleasantness of an image is not significantly influenced by the examined image global characteristics. These results emphasize the complexity of emotional responses to visual stimuli and suggest that different underlying mechanisms may govern arousal and valence.

Our findings align with existing research showing that color attributes significantly influence arousal. For example, Weijs et al. (2023) demonstrated that red environments increase physiological arousal compared to blue ones, with darker environments leading to higher arousal, indicated by increased heart rate and decreased heart rate variability. Similarly, Zieliński (2016) found that higher color saturation leads to stronger skin conductance responses. Duan et al. (2018) showed that yellow backgrounds are associated with low arousal states, while red backgrounds correspond to high arousal states, and orange backgrounds result in high impulsivity. This corroborates our identification of hue as a significant predictor of arousal. Additionally, research by Hooke et al. (1975) and Valdez and Mehrabian (1994) has explored the emotional effects of color, with Valdez and Mehrabian finding that saturation and brightness strongly affect emotions. They identified green-yellow, blue-green, and green as the most arousing colors, aligning with our findings on the influence of color attributes on arousal. However, our study did not find significant predictors for valence, suggesting that factors beyond the examined image characteristics may influence the pleasantness or unpleasantness of visual stimuli.

Research on color-emotion associations reveals that hues and chroma can influence emotional perceptions and preferences. For example, Moller et al. (2009) found that red is associated with failure and negative words, while green is linked to success. Increased chroma in images enhances perceptions of happiness, arousal, and positive valence. Natural content elicits more positive emotions than urban scenes, and green hues are less arousing than red ones. Color preferences may serve an adaptive function, with people tending to like colors associated with objects they find appealing. This ecological valence theory explains 80 percent of the variance in color preference ratings. Gender, expertise, culture, and perceptual experience also influence hue preferences. These findings illustrate the complex interplay between color dimensions, image content, and emotional responses, suggesting that color carries psychologically relevant meaning beyond mere aesthetics.

Studies by Suk and Irtel (2009) investigated how color attributes influence emotional dimensions of valence, arousal, and dominance using the Self-Assessment Manikin (SAM). They found that all three color attributes (hue, chroma, and luminance) affected emotional responses, with chroma consistently showing positive correlations with emotional dimensions. Suk (2006) expanded on this research, conducting four experiments to analyze color-emotion relationships. Both studies revealed that emotional responses to color vary more strongly with tone than hue categories. While Suk and Irtel (2009) compared responses between paper and CRT monitor presentations, Suk (2006) also examined color emotions in the context of other visual stimuli like pictures and film clips. These findings contribute to understanding how color influences emotion across different media and contexts, with potential applications in marketing and design.

Feature congestion has been identified as a measure of visual clutter in several studies by Rosenholtz et al. (2005, 2007). This measure is based on the concept that a more cluttered display makes it harder to add a new item that would draw attention. The feature congestion measure considers factors such as color, luminance contrast, and orientation. It has been shown to correlate well with subjective assessments of visual clutter and search performance in complex imagery. The researchers have explored its use as a substitute for set size in visual search models, demonstrating its applicability to various types of displays, including search-in-clutter tasks. Other measures of visual clutter, such as sub-band entropy and edge density, have also been investigated, but feature congestion appears to account for additional factors like color variability. Our identification of feature congestion as a significant predictor of arousal aligns with these findings, emphasizing the role of visual complexity in emotional responses. Our investigation into the relationship between participants' mood and their affective judgments of arousal and valence revealed no significant associations. Participants' mood and self-esteem were measured initially through self-reported attitudes and self-esteem levels. Analysis showed that neither participants' self-reported attitudes nor their self-esteem levels had a statistically significant impact on their mean arousal or valence scores. Specifically, one-way ANOVA tests indicated no significant differences in mean valence or arousal based on attitude or self-esteem ratings. These findings suggest that the emotional responses elicited by the visual stimuli were not significantly influenced by the participants' initial mood states. This indicates that the affective judgments of the images were robust across different initial mood conditions, underscoring the complexity of emotional processing and its relative independence from transient mood variations. Further research could explore additional factors that might moderate the relationship between mood and affective judgments.

We should expect to see various emotional reactions to images having semantic meaning. However, the way how such semantics-rich visual samples interact with our brain, and ultimately evoke reactions, is not fully understood. Therefore, in this study we used a generative mechanism that is more interpretable due to closer analogies between the generative process based on maximizing specific sections of Convolutional Neural Networks and the visual cortex of the brain. This has been demonstrated earlier in the case of monkeys (Bashivan et al., 2019) but, to our knowledge, has never been researched with human subjects. Moreover, these semantics-free “deep dream” images should limit a bias that could be observed in the responses due to subjects' experience and memories related to naturalistic images.

Overall, the findings offered in this study may have significant implications for the design of visual stimuli in various fields such as marketing, virtual reality, and human-computer interaction. Understanding which image characteristics influence arousal can help in creating more engaging content that captures attention and elicits desired emotional responses. Additionally, this research paves the way for developing tools aimed at brain stimulation and supporting wellbeing. By identifying specific visual features that influence emotional arousal, we can create applications designed to improve mental health and assist patients with certain mental conditions. These tools could use tailored visual stimuli to manage stress, enhance mood, and support therapeutic interventions.

### 5.1 Limitations and future research

This study is the first, known to us, that measures emotional reactions evoked by images synthesized by deep learning-based models and reports on correlations between these reactions and areas (layers) of a neural network mimicking the way the human visual system works. However, this study has, of course, a few limitations, which provide a good set of ideas for immediate future work discussed in this closing section.

One limitation of this study is the reliance on self-reported data, which serves as a proxy for a true brain reaction and thus may be subject to biases. Another limitation is the sample size, both related to the number of subjects and to the number of random noise seeds used to synthesize samples for each layer (five in this study). Subjects' diversity may limit the generalization of findings, and stochasticity of the synthesis process may result in generated samples that are outliers, which in consequence may reduce the replicability of this study. Finally, this study is limited to visual stimuli, but analogous reactions may be evoked when presenting synthesized audio or haptic stimuli to bring more senses into play.

Future research should consider using more objective measures of arousal and valence. These measures can include basic physiological responses (blood pressure, blood volume changes, respiration, perspiration/skin conductance, pupil dynamics, or face micro- and macro-movements) or—if possible—involve direct measurements of brain activity via functional magnetic resonance imaging (fMRI). In addition, extending these studies with a more diverse and larger participant pool would enable more authoritative conclusions on possible connections between artificial neural network-based models and processes governing human emotional reactions. Furthermore, our immediate research plans include immersing the participants into a Virtual Reality world with synthesized visual stimuli (via a Virtual Reality headset) which would allow the participants to walk closer to and further from images and thus change the scale and homomorphic transformations of pictures, which in consequence may stimulate the brain in various ways. This paradigm will address the effect of feature scale on activations of visual neurons, and will replace the now-arbitrary scale used in this current and many other studies. Finally, we also aim to explore the combination of multi-modal stimuli (visual, audio, and haptic) and their combined effects on mood and self-esteem, measured both before and after exposure to such stimuli. This study would provide insights into the design of an effective and affordable tool to evoke changes in emotional states resulting from interaction with precisely synthesized multi-modal stimuli.

## Data Availability

The dataset of – all visual stimuli synthesized for this work, and– subjects' responses, recorded via the Prolific and appropriately anonymized (e.g., with the Prolific worker IDs stripped off from the data) is made available along with this paper. To obtain a copy of the dataset, interested researchers need to complete and submit the data sharing license agreement available at: https://cvrl.nd.edu/projects/data (Emotional Response to DeepDream Pictures dataset). The source codes allowing all interested researchers to fully replicate the image synthesis process are available via the following GitHub repository https://github.com/CVRL/human-reactions-to-deepdream-samples.

## Footnotes

^1^ https://www.lockluck.eu

^2^ www.prolific.com
