# Supplementary material for: Assessment of human emotional reactions to visual stimuli “deep-dreamed” by artificial neural networks
Source: Front Psychol. 2024 Dec 24;15:1509392. doi: 10.3389/fpsyg.2024.1509392 (PMC11703666; doi:10.3389/fpsyg.2024.1509392)
Supplement: Supplementary file 1 [file Data_Sheet_1.PDF]

Supplementary Material

1 INCEPTION V3 MODEL

Figure S1 presents the structure of the Inception v3 model proposed by Szegedy et al. (2016) used in this study. Color-coded shapes are used to differentiate types of layers present in that model. The names of these layers are kept as in the original work, and are used consequently in the main paper when referring to a concrete part of the network.

2 VALENCE-AROUSAL COMPLETE HEATMAPS

Joint distributions of valence (vertical axis) and arousal (horizontal axis) responses from all subjects (smoothed to create heat maps for better visibility). Central point corresponds to neutral reactions. Left and bottom (right and top) shifts from the central points correspond to negative (positive) valence and arousal, respectively. White cross and red circle markers represent the mean and median values of all responses, respectively.

Figures S5 – S12 show pictures generated for Inception v3 layers which obtained various combinations of extreme median arousal and valence values, *e.g.*, the smallest average arousal and valence (Fig. S5), or the largest average arousal and valence (Fig. S9).

3 LAYER IMAGE CHARACTERISTICS

Tables S1 – S3 present mean values of various image metrics calculated over five instances of synthesized images for each Inception v3 layer.

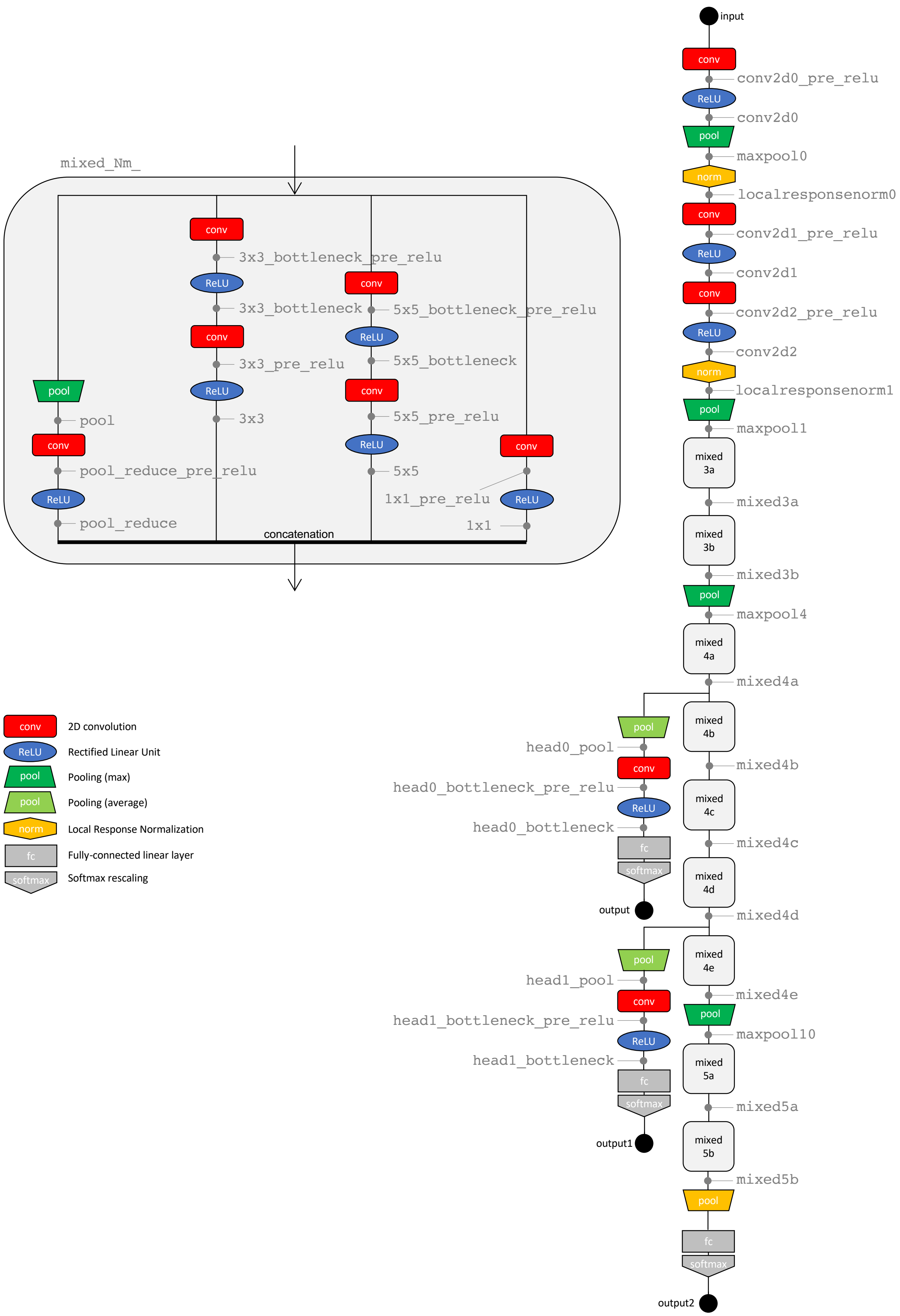

Figure S1. Inception v3 model used in this study.

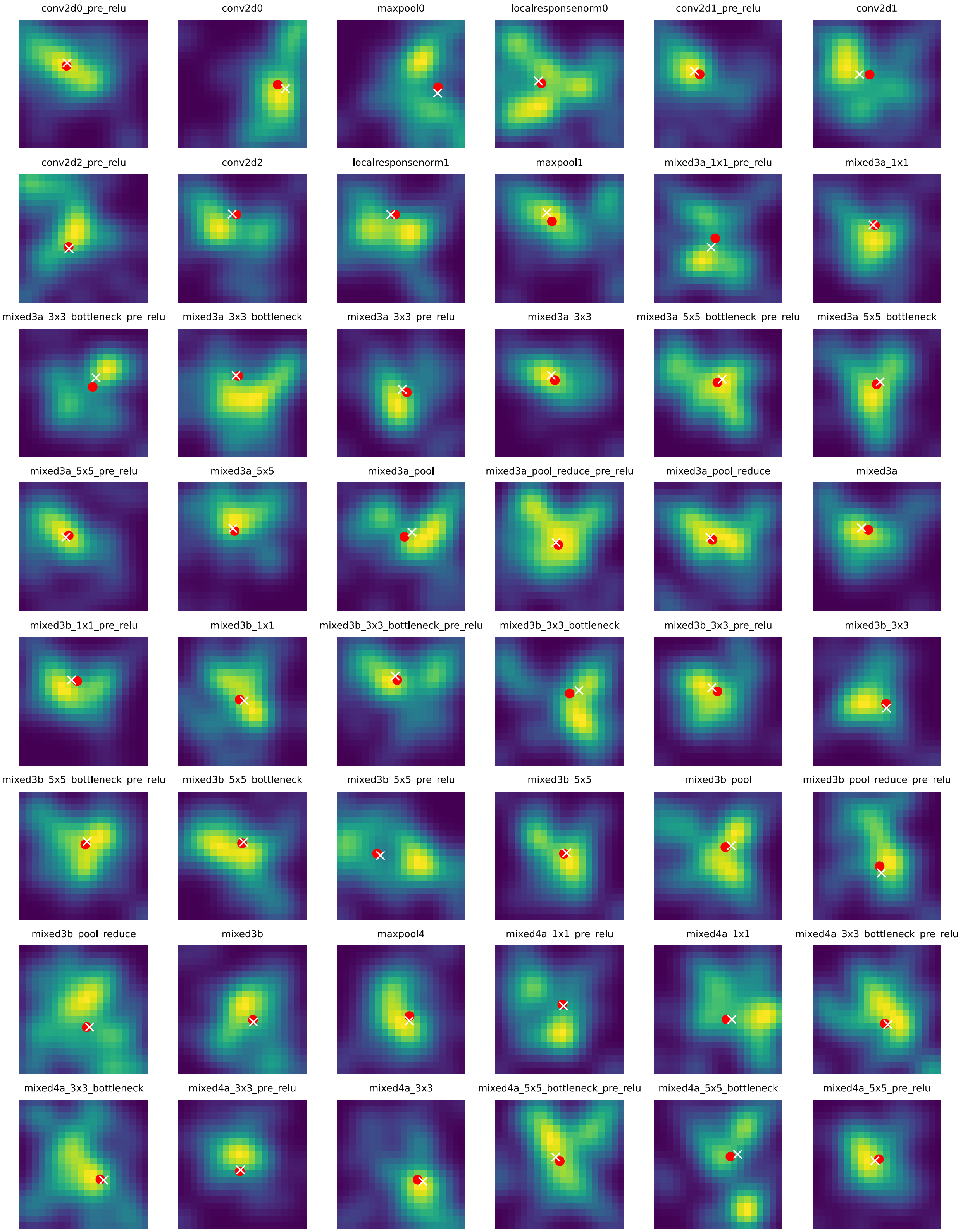

Figure S2. Joint distributions of valence (vertical axis) and arousal (horizontal axis) responses from all subjects obtained for the first 48 layers of the Inception v3 model shown in Fig. S1. The remaining layers are visualized in Figs. S3 and S4).

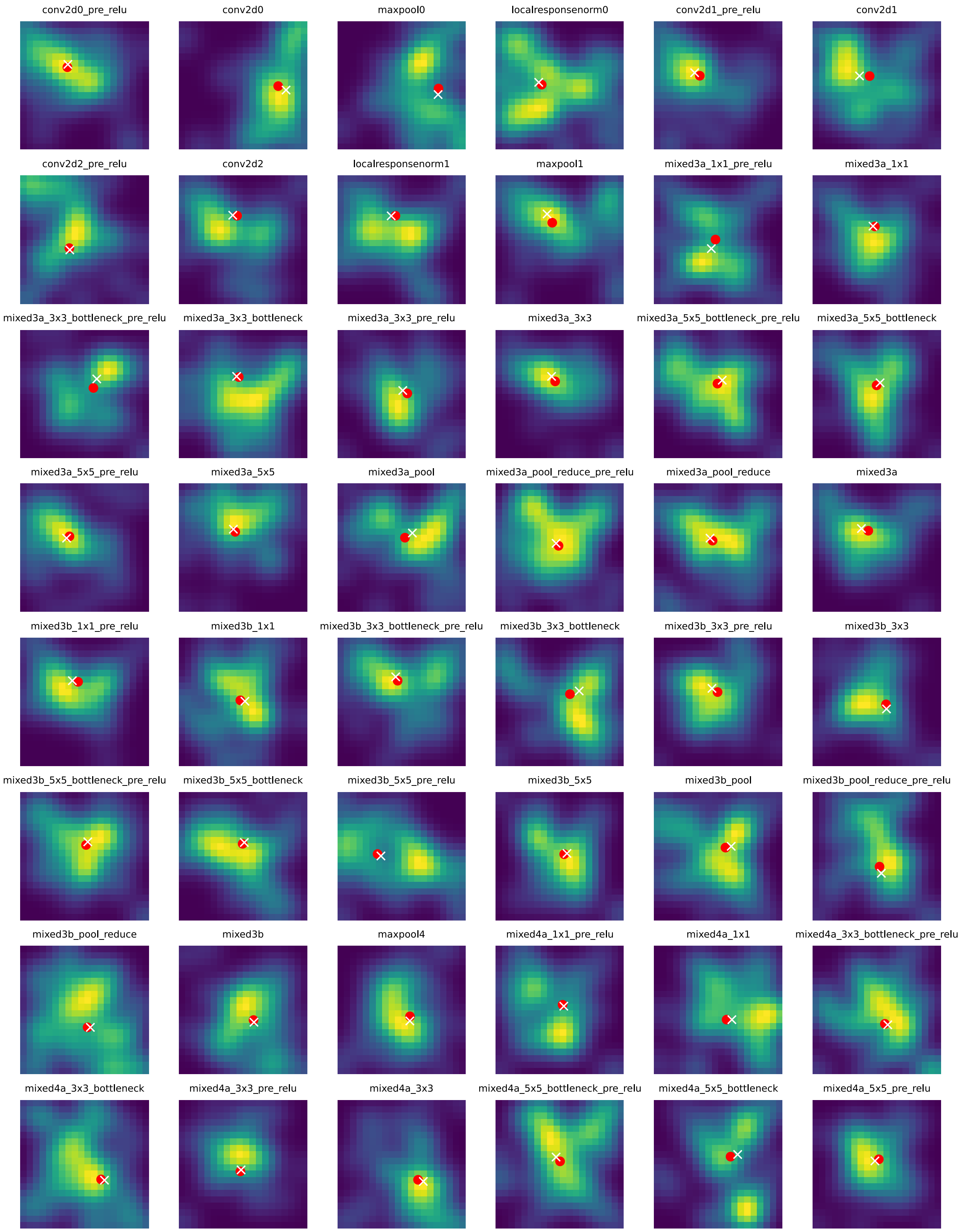

Figure S3. Same as in Fig. S2 except that heat maps for the middle 48 layers are shown.

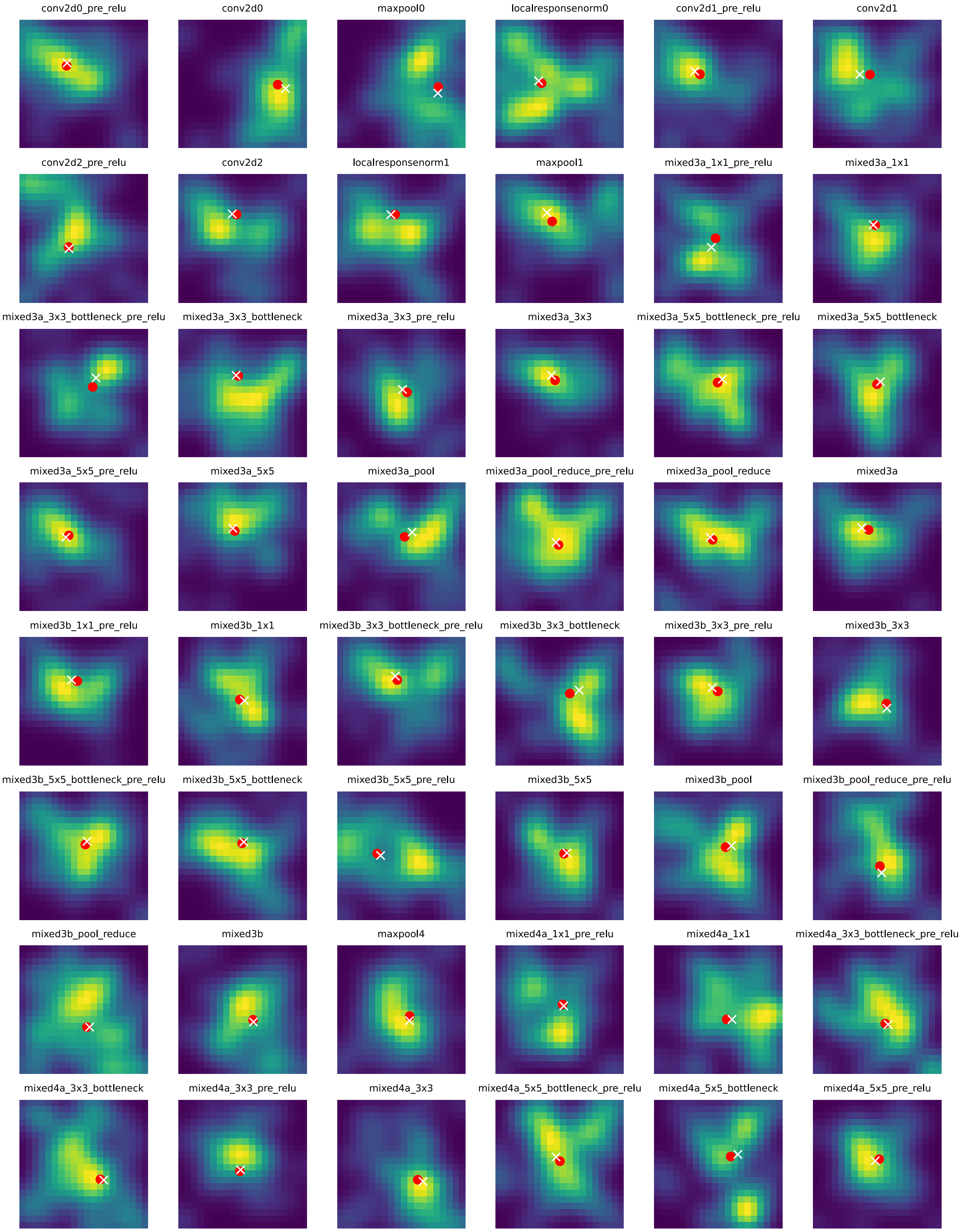

Figure S4. Same as in Fig. S2 except that heat maps for the last 48 layers are shown.

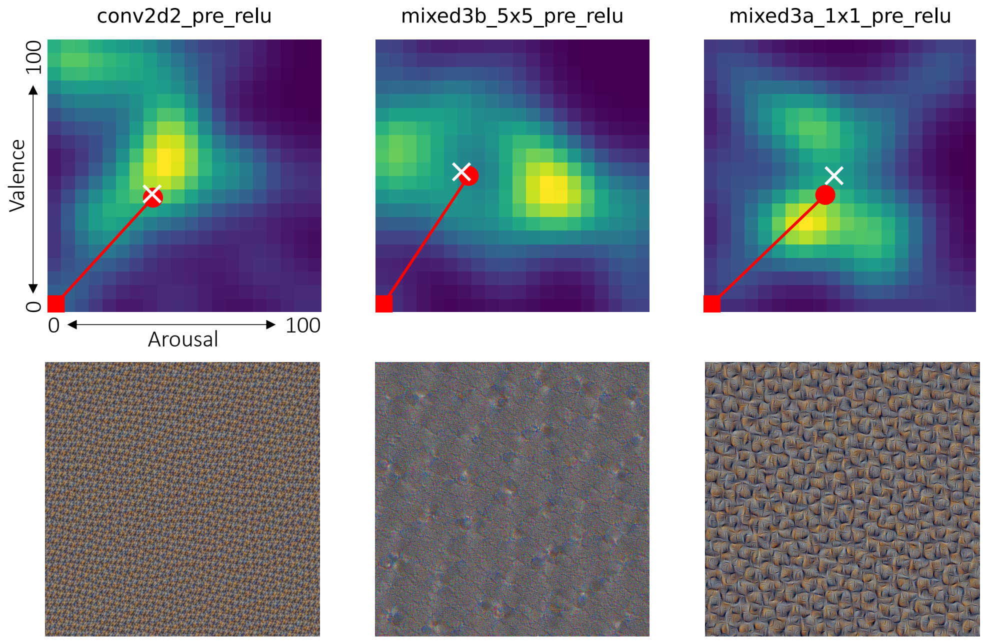

Figure S5. Visualization of pictures generated for three layers which obtained the smallest average arousal and valence scores provided by the participants. As in Figs S2–S4, central point corresponds to neutral reactions, left and bottom (right and top) shifts from the central points correspond to negative (positive) valence and arousal, respectively, and white cross and red circle markers represent the mean and median values of all responses, respectively.

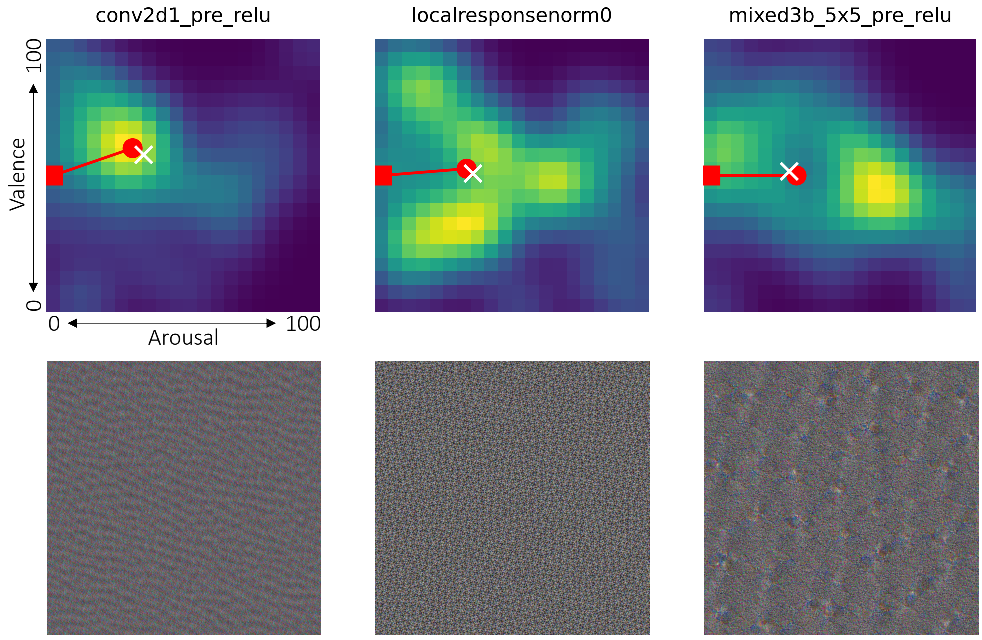

Figure S6. Same as in Fig. S5, except that three synthesized images are shown that generated reactions closest to the **neutral (50) valence** and **negative (0) arousal**.

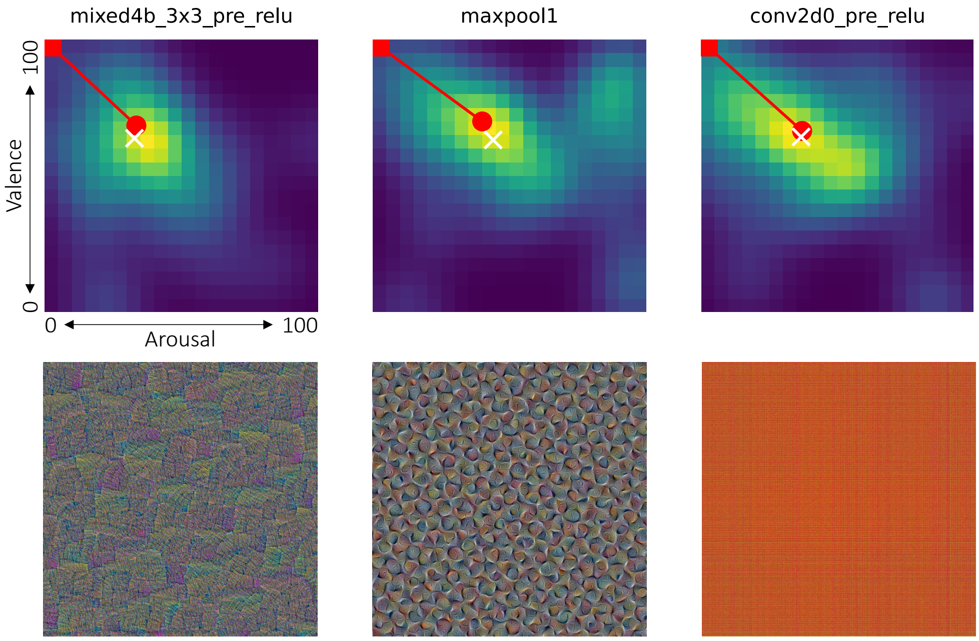

Figure S7. Same as in Fig. S5, except that three synthesized images are shown that generated reactions closest to the **positive (100) valence** and **negative (0) arousal**.

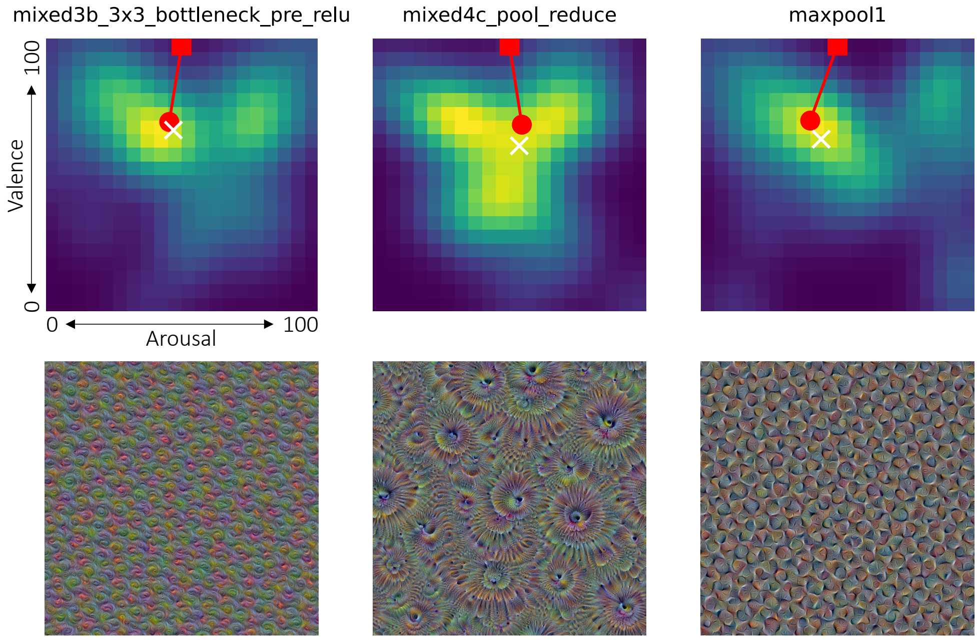

Figure S8. Same as in Fig. S5, except that three synthesized images are shown that generated reactions closest to the **positive (100) valence** and **neutral (50) arousal**.

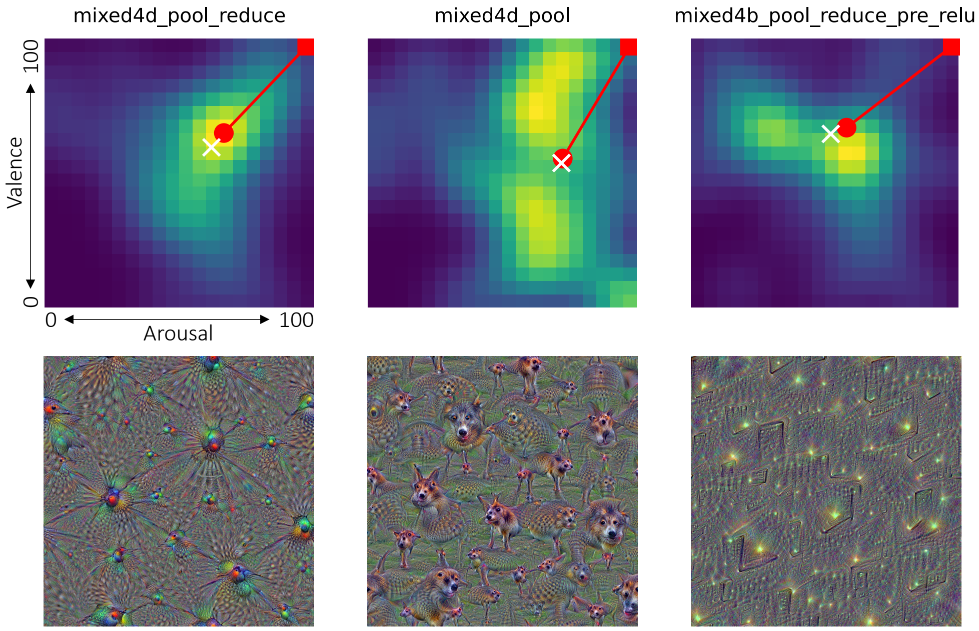

Figure S9. Same as in Fig. S5, except that three synthesized images are shown that generated reactions closest to the **positive (100) valence** and **positive (100) arousal**.

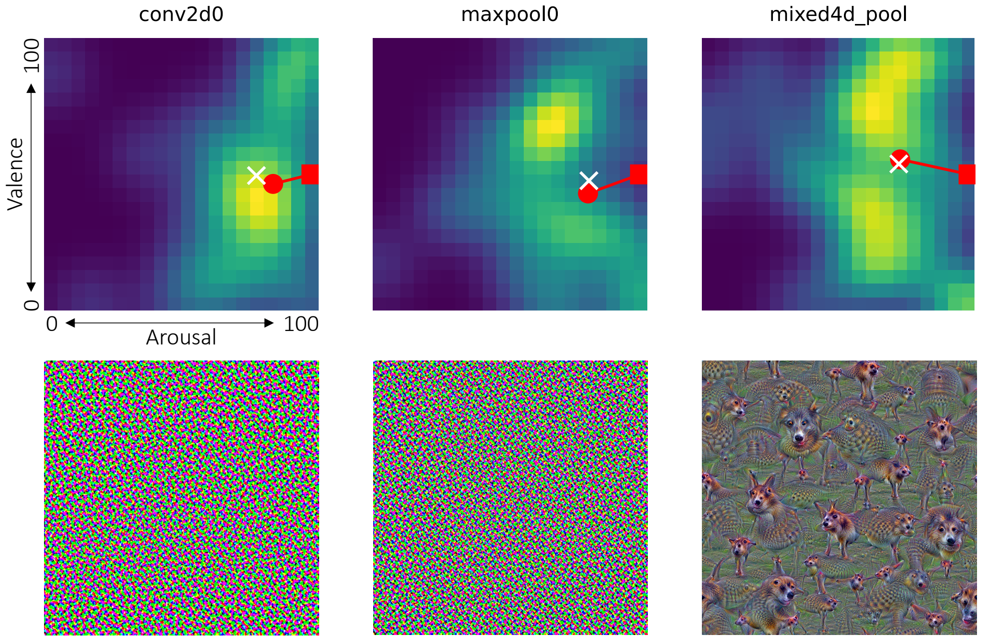

Figure S10. Same as in Fig. S5, except that three synthesized images are shown that generated reactions closest to the **neutral (50) valence** and **positive (100) arousal**.

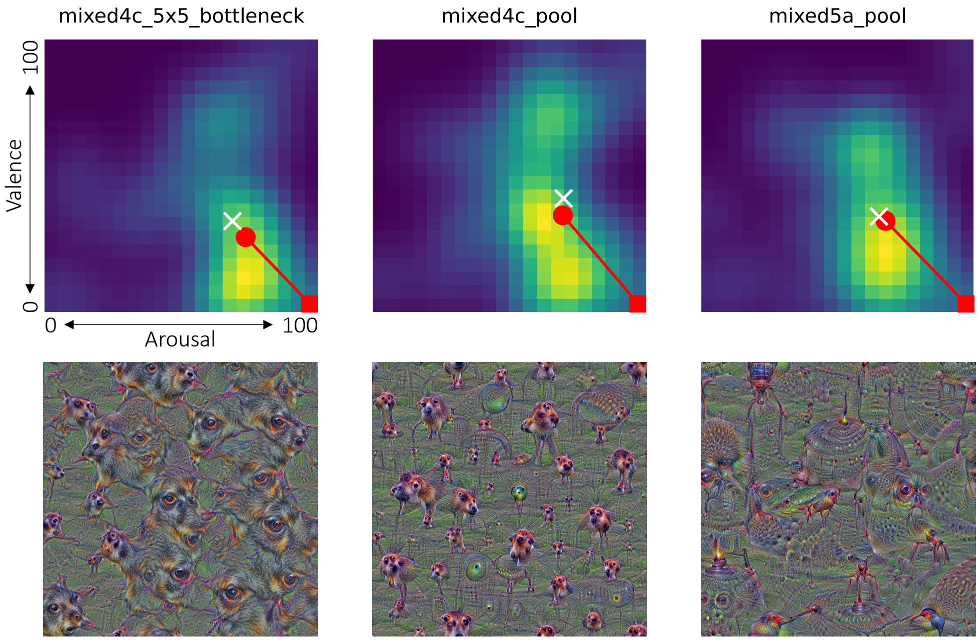

Figure S11. Same as in Fig. S5, except that three synthesized images are shown that generated reactions closest to the **negative (0) valence** and **positive (100) arousal**.

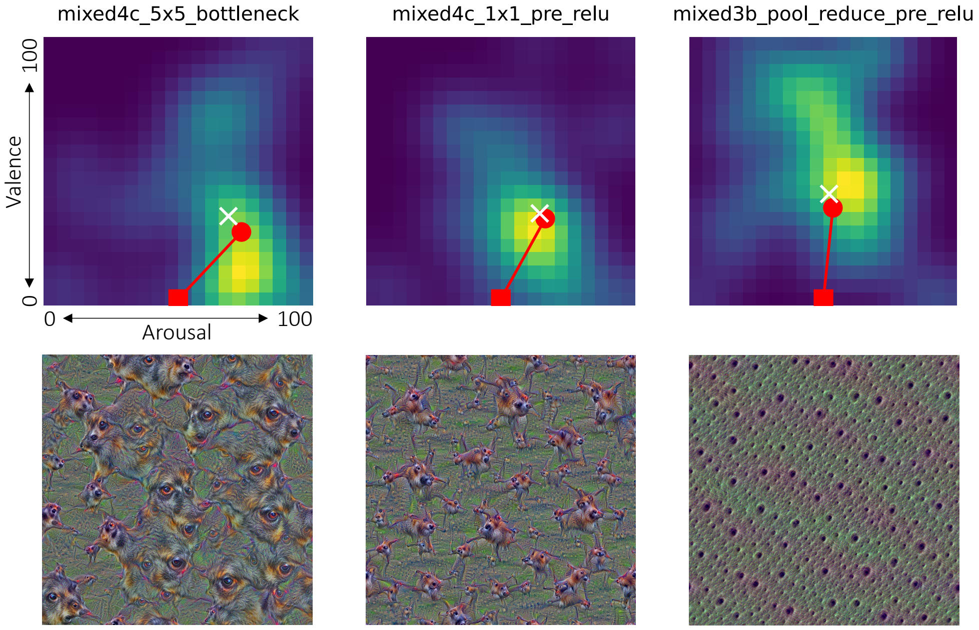

Figure S12. Same as in Fig. S5, except that three synthesized images are shown that generated reactions closest to the **negative (0) valence** and **neutral (50) arousal**.

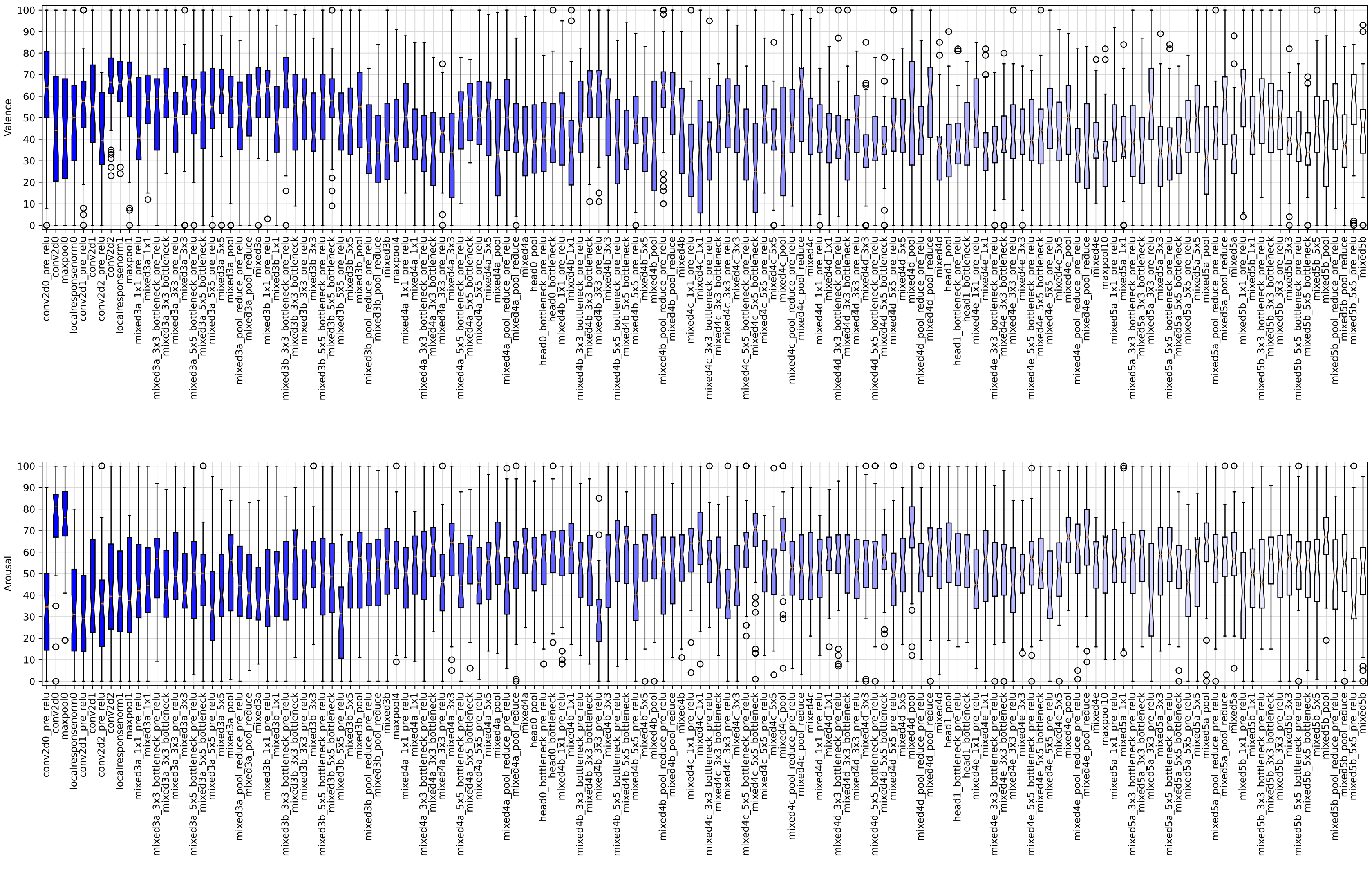

Figure S13. Boxplots illustrating the valence (top plot) and arousal (bottom plot) scores sorted by the layer's position in the Inception network. The formatting of boxplots is the same as in Fig. 6 in the main manuscript. The colors of boxplots correspond to the depth of a given layer: light-shaded boxplots correspond to deeper layers, while darker color represents layers closer to the input. This color schema was applied to Fig. 10 in the main manuscript so that plots in Figures 10 and S13 could be analyzed together.

Table S1. Mean values of image metrics calculated over five instances of synthesized images for each Inception v3 layer.

Layer	Valence	Arousal	Hue	Saturation	Brightness	JPEG %	Congestion	Entropy
conv2d0	46.47	74.65	185.74	187.11	189.51	65.55	202.16	5.13
conv2d0_pr	61.80	34.28	145.58	139.21	138.15	28.42	45.70	5.12
conv2d1	54.88	41.85	110.09	111.07	110.15	35.89	61.68	4.65
conv2d1_pr	54.07	32.57	114.14	107.39	106.48	35.53	54.89	5.21
conv2d2	66.18	43.45	98.63	97.86	98.00	35.92	55.80	4.52
conv2d2_pr	41.04	35.15	86.58	88.23	88.89	32.23	55.04	5.28
head0_bn	42.43	60.33	98.90	98.49	98.67	36.93	63.18	4.50
head0_bn_pr	40.83	56.77	98.58	99.42	99.61	37.06	63.20	4.47
head0_pool	40.13	54.66	96.28	95.08	95.75	35.31	60.04	4.35
head1_bn	40.34	55.93	104.50	103.05	102.93	38.43	66.78	4.53
head1_bn_pr	35.79	56.06	104.99	102.64	102.54	38.92	67.37	4.54
head1_pool	34.81	60.10	98.15	97.72	98.30	36.42	63.54	4.60
localrespnorm0	48.22	32.74	115.46	104.93	104.17	46.21	88.53	5.35
localrespnorm1	65.68	42.81	96.41	96.00	96.00	34.17	52.98	4.51
maxpool0	44.51	74.58	181.13	184.12	187.52	69.85	214.91	5.16
maxpool1	60.91	42.09	90.29	90.66	90.27	32.08	63.36	4.57
maxpool10	31.63	55.38	100.57	99.95	100.41	36.75	59.54	4.35
maxpool4	42.25	54.12	96.26	95.81	96.24	31.61	53.06	4.30
mxd3a	60.79	40.39	90.97	92.09	92.05	35.64	61.00	4.54
mxd3a_1x1	57.78	45.42	90.05	90.10	88.16	33.40	63.26	4.49
mxd3a_1x1_pr	47.57	44.76	89.10	88.77	88.44	33.61	69.26	4.48
mxd3a_3x3	57.44	43.79	95.33	96.38	96.71	36.27	63.75	4.30
mxd3a_3x3_bn	60.50	44.51	97.77	96.53	96.77	37.15	65.53	4.57
mxd3a_3x3_bn_pr	52.22	55.46	101.59	100.17	99.43	35.82	57.88	4.77
mxd3a_3x3_pr	47.71	51.18	97.69	98.27	98.89	34.12	45.42	4.99
mxd3a_5x5	59.92	41.45	97.35	97.60	98.08	29.56	53.57	4.88
mxd3a_5x5_bn	53.65	46.66	102.13	102.51	102.14	27.04	47.37	4.74
mxd3a_5x5_bn_pr	54.68	44.98	96.90	97.38	97.47	30.26	68.03	5.07
mxd3a_5x5_pr	56.08	35.76	93.56	91.70	91.12	35.81	64.69	4.65
mxd3a_pool	55.38	50.96	92.70	93.06	91.89	29.74	55.12	4.21
mxd3a_pool_rdc	52.00	42.84	100.57	100.17	100.45	36.21	58.02	4.69
mxd3a_pool_rdc_pr	49.06	46.42	99.91	100.26	99.97	36.60	61.87	4.61
mxd3b	39.13	55.68	92.81	92.06	93.03	33.00	61.56	4.60
mxd3b_1x1	48.70	45.41	92.36	92.30	91.52	35.44	51.39	4.54
mxd3b_1x1_pr	63.12	43.55	93.44	93.16	93.00	31.12	67.90	4.78
mxd3b_3x3	45.57	54.67	95.76	94.68	94.95	33.84	53.87	4.69
mxd3b_3x3_bn	54.29	54.76	92.08	92.19	92.69	37.35	56.28	4.50
mxd3b_3x3_bn_pr	64.19	44.51	97.75	97.12	96.98	33.95	56.33	4.62
mxd3b_3x3_pr	55.14	46.67	102.09	100.19	100.33	34.31	48.75	4.80
mxd3b_5x5	48.88	50.58	96.79	97.22	97.41	34.97	46.64	4.70
mxd3b_5x5_bn	56.98	48.38	92.35	91.89	91.91	32.52	58.76	4.52
mxd3b_5x5_bn_pr	55.92	47.94	93.47	92.68	92.64	30.97	62.23	4.87
mxd3b_5x5_pr	49.00	28.86	106.88	103.51	103.44	37.03	54.05	4.42
mxd3b_pool	53.60	53.00	94.39	94.57	94.48	32.21	58.18	4.24
mxd3b_pool_rdc	33.84	50.27	91.58	92.15	92.54	33.54	54.69	4.60
mxd3b_pool_rdc_pr	38.16	49.79	94.94	94.12	93.56	35.05	56.18	4.71
mxd4a	38.21	61.13	94.64	94.59	94.99	34.40	55.60	4.42
mxd4a_1x1	40.50	53.90	96.83	97.35	97.39	33.17	57.40	4.51
mxd4a_1x1_pr	51.61	49.16	99.12	98.23	98.38	33.92	56.80	4.19
mxd4a_3x3	35.26	60.40	95.04	94.27	94.79	33.67	54.04	4.13
mxd4a_3x3_bn	35.57	60.39	95.63	96.21	96.19	33.21	57.27	4.34
mxd4a_3x3_bn_pr	35.29	54.02	93.90	93.97	94.99	31.83	63.18	4.94
mxd4a_3x3_pr	42.16	45.92	104.81	102.04	101.43	37.88	55.13	4.21
mxd4a_5x5	48.75	52.83	98.21	97.77	98.77	35.25	55.37	4.20
mxd4a_5x5_bn	52.12	56.74	95.70	94.94	94.85	32.81	60.83	4.57
mxd4a_5x5_bn_pr	50.02	47.67	97.17	97.09	96.96	32.89	66.90	4.78

Table S2. Mean values of image metrics continued.

Layer	Valence	Arousal	Hue	Saturation	Brightness	JPEG %	Congestion	Entropy
mxd4a_5x5_pr	51.24	48.44	100.84	100.33	99.82	37.71	57.56	4.33
mxd4a_pool	35.98	59.80	96.05	96.87	97.45	32.23	54.92	4.29
mxd4a_pool_rdc	43.75	54.19	94.41	94.52	94.87	34.42	57.21	4.31
mxd4a_pool_rdc_pr	52.23	45.84	100.70	99.28	99.75	37.79	62.94	4.62
mxd4b	45.91	56.74	95.67	96.13	95.79	36.45	58.74	4.34
mxd4b_1x1	36.23	61.46	96.91	95.92	95.91	34.45	59.35	4.38
mxd4b_1x1_pr	45.82	60.21	97.48	96.56	96.44	35.00	65.46	4.69
mxd4b_3x3	50.57	52.35	97.14	97.48	97.84	37.01	62.73	4.48
mxd4b_3x3_bn	60.81	50.98	97.27	97.18	97.47	37.06	65.27	4.47
mxd4b_3x3_bn_pr	45.78	52.22	94.22	94.50	95.31	36.49	66.76	4.88
mxd4b_3x3_pr	60.52	30.33	105.77	102.72	102.16	39.75	56.84	4.29
mxd4b_5x5	37.13	57.51	98.79	99.56	99.87	33.91	58.96	4.37
mxd4b_5x5_bn	41.87	58.75	93.79	92.88	92.87	34.48	59.00	4.43
mxd4b_5x5_bn_pr	38.37	58.84	95.27	95.09	95.27	35.24	66.00	4.86
mxd4b_5x5_pr	48.60	43.61	105.72	103.27	103.16	38.28	63.83	4.34
mxd4b_pool	42.10	61.62	96.30	96.34	96.85	33.57	57.34	4.33
mxd4b_pool_rdc	54.93	51.45	97.77	96.76	97.37	36.53	63.32	4.36
mxd4b_pool_rdc_pr	62.55	49.63	102.32	100.50	99.83	37.18	62.83	4.36
mxd4c	47.24	53.48	94.91	95.18	95.29	35.70	59.83	4.26
mxd4c_1x1	36.39	65.67	96.43	96.36	96.07	34.66	61.01	4.35
mxd4c_1x1_pr	31.94	62.13	96.28	94.84	95.01	35.54	62.10	4.47
mxd4c_3x3	48.65	48.66	94.50	95.09	95.51	36.43	61.81	4.46
mxd4c_3x3_bn	45.09	49.12	96.54	96.21	96.50	35.87	62.70	4.62
mxd4c_3x3_bn_pr	36.81	56.00	95.57	94.04	94.25	35.57	64.69	4.92
mxd4c_3x3_pr	53.22	40.94	106.18	103.84	103.43	39.13	58.42	4.34
mxd4c_5x5	41.11	51.20	96.10	95.66	94.69	36.14	57.91	4.50
mxd4c_5x5_bn	31.35	66.18	93.67	94.26	94.14	34.60	62.56	4.48
mxd4c_5x5_bn_pr	35.43	60.08	95.75	94.78	95.80	34.62	66.39	4.59
mxd4c_5x5_pr	50.83	52.73	101.63	100.15	100.36	38.14	61.23	4.53
mxd4c_pool	38.49	67.25	98.06	97.57	97.20	34.61	60.02	4.23
mxd4c_pool_rdc	58.14	51.06	96.92	96.37	97.74	34.96	60.31	4.31
mxd4c_pool_rdc_pr	46.90	48.20	99.36	98.92	98.67	37.65	65.04	4.51
mxd4d	32.58	57.08	96.43	96.84	96.51	36.00	59.61	4.60
mxd4d_1x1	40.08	58.12	98.51	98.85	100.09	35.14	63.39	4.60
mxd4d_1x1_pr	42.23	51.45	102.78	100.45	100.33	37.24	60.33	4.75
mxd4d_3x3	33.74	56.06	100.29	99.69	99.22	36.77	63.59	4.58
mxd4d_3x3_bn	36.06	58.30	95.08	95.45	95.62	35.31	64.07	4.55
mxd4d_3x3_bn_pr	41.85	56.20	100.25	98.79	98.95	36.89	67.43	4.78
mxd4d_3x3_pr	45.67	50.58	106.82	103.66	102.96	39.78	60.42	4.70
mxd4d_5x5	43.06	53.45	97.13	96.87	97.21	36.53	60.69	4.65
mxd4d_5x5_bn	37.12	55.40	97.08	96.31	96.32	35.71	61.54	4.74
mxd4d_5x5_bn_pr	37.92	56.79	97.47	97.36	96.83	35.63	66.62	4.68
mxd4d_5x5_pr	45.06	47.21	102.72	100.50	100.28	39.10	62.42	4.60
mxd4d_pool	49.20	69.51	96.97	97.51	96.93	34.91	60.32	4.25
mxd4d_pool_rdc	57.38	59.40	100.88	99.99	100.60	36.12	63.68	4.41
mxd4d_pool_rdc_pr	42.65	53.27	104.73	102.50	103.23	39.22	66.57	4.67
mxd4e	39.49	52.19	98.15	96.92	97.25	37.03	62.98	4.66
mxd4e_1x1	35.53	56.31	97.54	97.06	97.06	36.70	65.17	4.75
mxd4e_1x1_pr	49.92	47.75	100.51	99.25	98.55	37.67	61.70	4.67
mxd4e_3x3	41.68	49.05	98.11	97.50	98.24	37.50	62.08	4.60
mxd4e_3x3_bn	40.52	52.17	96.56	96.87	97.28	36.22	64.41	4.67
mxd4e_3x3_bn_pr	36.60	50.12	97.51	96.45	96.74	36.44	65.91	4.51
mxd4e_3x3_pr	42.60	45.57	100.86	100.39	100.22	37.76	61.24	4.77
mxd4e_5x5	42.06	52.98	97.35	96.93	97.30	36.61	61.54	4.73
mxd4e_5x5_bn	43.52	53.13	96.81	97.00	97.67	36.31	63.23	4.69
mxd4e_5x5_bn_pr	41.63	54.02	97.65	96.79	97.11	36.32	68.51	4.93
mxd4e_5x5_pr	50.08	43.13	106.86	104.51	103.51	39.94	63.61	4.71

Table S3. Mean values of image metrics continued.

Layer	Valence	Arousal	Hue	Saturation	Brightness	JPEG %	Congestion	Entropy
mxd4e_pool	45.61	65.56	99.34	99.30	99.22	35.73	62.31	4.37
mxd4e_pool_rdc	34.16	65.58	99.28	98.45	98.30	36.78	63.06	4.58
mxd4e_pool_rdc_pr	33.81	59.49	100.33	99.61	100.59	37.44	63.26	4.68
mxd5a	33.38	56.98	100.35	99.61	99.85	36.72	63.74	4.50
mxd5a_1x1	37.77	55.51	101.02	100.70	100.46	37.08	64.26	4.62
mxd5a_1x1_pr	41.42	57.28	103.32	100.51	100.81	37.77	64.25	4.52
mxd5a_3x3	33.51	58.29	100.39	100.25	99.74	36.87	64.14	4.48
mxd5a_3x3_bn	32.91	61.45	98.84	98.41	98.37	36.96	62.26	4.52
mxd5a_3x3_bn_pr	38.62	56.87	101.99	101.06	100.85	36.99	69.56	4.85
mxd5a_3x3_pr	53.47	44.12	110.57	106.13	106.59	40.49	62.70	4.65
mxd5a_5x5	47.64	51.19	102.04	100.61	100.46	36.89	63.18	4.59
mxd5a_5x5_bn	37.28	51.70	99.79	99.28	99.28	36.72	63.28	4.65
mxd5a_5x5_bn_pr	34.88	58.92	99.38	98.42	97.90	36.82	66.60	4.69
mxd5a_5x5_pr	40.67	45.98	109.07	105.45	104.22	38.70	62.61	4.54
mxd5a_pool	32.37	62.88	98.53	97.61	97.63	36.49	63.41	4.42
mxd5a_pool_rdc	53.69	57.27	100.76	100.48	100.42	36.89	65.47	4.26
mxd5a_pool_rdc_pr	42.62	54.10	104.97	103.75	102.32	38.49	67.24	4.37
mxd5b	44.85	49.45	103.05	100.38	99.75	38.65	68.27	4.50
mxd5b_1x1	45.37	46.50	104.67	102.72	101.84	39.10	72.42	4.84
mxd5b_1x1_pr	57.33	40.89	110.42	106.11	105.61	42.20	62.56	4.47
mxd5b_3x3	39.45	54.32	101.62	101.10	101.21	38.02	72.03	4.82
mxd5b_3x3_bn	50.08	55.32	98.42	97.93	97.22	36.23	68.30	4.40
mxd5b_3x3_bn_pr	56.12	49.05	109.57	105.52	105.42	41.67	71.38	4.72
mxd5b_3x3_pr	47.04	55.24	107.69	104.28	104.17	40.75	64.57	4.48
mxd5b_5x5	43.92	53.33	108.49	104.61	103.92	39.51	64.83	4.46
mxd5b_5x5_bn	34.40	53.56	101.98	100.45	100.02	36.87	69.50	4.61
mxd5b_5x5_bn_pr	38.15	54.56	102.34	100.81	101.20	37.04	72.58	4.79
mxd5b_5x5_pr	54.58	41.12	107.14	103.36	103.04	41.54	68.67	4.50
mxd5b_pool	37.33	66.57	103.25	102.92	102.34	36.42	63.76	4.36
mxd5b_pool_rdc	37.94	52.83	103.42	100.92	100.96	38.73	68.33	4.51
mxd5b_pool_rdc_pr	52.17	48.44	107.74	104.39	104.40	41.26	71.89	4.74

REFERENCES

Szegedy, C., Vanhoucke, V., Ioffe, S., Shlens, J., and Wojna, Z. (2016). Rethinking the inception architecture for computer vision. In *2016 IEEE Conference on Computer Vision and Pattern Recognition (CVPR)*. 2818–2826. doi:10.1109/CVPR.2016.308
